# Comparative Analysis of Platelet-Derived Extracellular Vesicle Protein Extraction Methodologies for Mass Spectrometry

**DOI:** 10.1021/acs.jproteome.5c00089

**Published:** 2025-07-21

**Authors:** Carmen Ráez-Meseguer, Andreu Miquel Amengual-Tugores, Maria Antònia Forteza-Genestra, Francisca Orvay-Pintos, Rosa M. Gomila, Gabriel Martorell-Crespí, Javier Calvo, Antoni Gayà, Marta Monjo, Joana Maria Ramis

**Affiliations:** † Group of Cell Therapy and Tissue Engineering (TERCIT), Research Institute on Health Sciences (IUNICS), 16745 University of the Balearic Islands (UIB), Palma 07122, Spain; ‡ Health Research Institute of the Balearic Islands (IdISBa), Palma 07120, Spain; § Department of Fundamental Biology and Health Sciences, UIB, Palma 07122, Spain; ∥ Scientific Technical Service (SCT), UIB, Palma 07122, Spain; ⊥ 203261 Fundació Banc de Sang i Teixits de les Illes Balears (FBSTIB), Palma 07004, Spain

**Keywords:** mass spectrometry, proteins, proteomics, methodologies, extracellular vesicles, platelets

## Abstract

The aim of this study is to present a comparative study of different methodologies for the extraction of proteins from platelet-derived extracellular vesicles (pEVs) prior to subsequent mass spectrometry (MS) analysis. pEVs were isolated by size exclusion chromatography (SEC) from human platelet lysates (PL) and characterized by identifying specific markers by Western blot, visualizing morphology by transmission electron microscopy (TEM) and analyzing concentration and size via nanoparticle tracking analysis (NTA). Protein isolation was performed through three different methodologies based on SDS-polyacrylamide gel electrophoresis (SDS-PAGE), organic solvent precipitation (OSP), or magnetic beads (MB), followed by protein digestion and sample acquisition by LC–MS/MS. Clustering of the samples according to methodology is observed in the principal component analysis (PCA), although no significant differences in terms of normalized abundances are reached. Similarly, a small number of proteins were identified as unique by each methodology, with 91.3% coincidence among all three procedures. In addition, the bioinformatic results of the enrichment analysis and the numbers of proteins already identified in the Vesiclepedia database are highly similar for the three methodologies. Overall, all three methodologies analyzed are optimal for the extraction of proteins from pEV and could be considered according to their intrinsic characteristics, in accordance with the research requirements.

## Graphical abstract

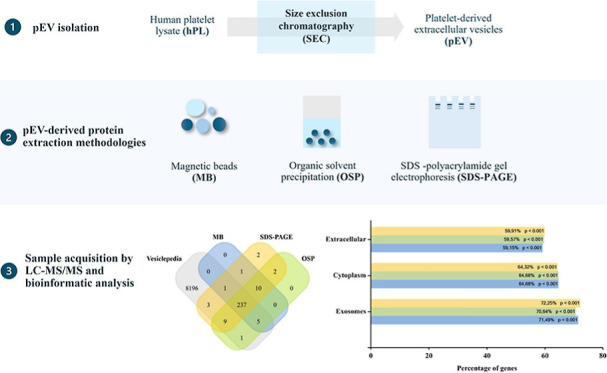

## Introduction

Extracellular vesicles (EV) have gained significant attention in the last decades due to their multiple properties.^1^ These nanometer-sized vesicles with lipidic membrane are released by different cell types and can be detected in a variety of biofluids.^2^ The molecular cargo of EVs closely reflects the cell type of origin^3^ with lipids, nucleic acids, and a variety of proteins, being the main molecules enriching the vesicular content.^4^ The EVs’ importance rest on their ability to transfer information in a protected manner to distant locations from the vesicular origin^5^ and to interact dynamically with other cells, contributing to cell-to-cell communication. Thus, because of their role in the regulation of physiological and pathological processes, circulating EVs are constantly evolving, reflecting multiple biological processes occurring in the organism.^6,7^

At present, EVs obtained from blood samples are being further investigated as potential biomarkers,^8^ as it is possible to detect specific EV characteristics, including size, concentration, or content, from the most abundant sample acquired in the clinical field.^9^ Hence, platelet-derived EVs (pEVs) are of particular interest given their potent signaling functions, which are increasingly being elucidated.^10^ Over time, the multifunctionality of pEVs has been demonstrated, considering that they can diffuse through tissues inaccessible to platelets.^11^ Consequently, pEVs as well as other platelet preparations, such as human platelet lysate (PL) or platelet-rich-plasma (PRP) are emerging as a potential source for regenerative medicine and provide numerous therapeutic benefits in the field.^12,13^ However, due to their intrinsic characteristics and involvement in modulating cell-to-cell signaling, pEVs have emerged as a superior alternative to other platelet concentrates.^14^ Several studies have proven the efficacy of pEVs enhancing wound healing,^15^ osteoinduction,^16^ hemostasis,^17^ and angiogenesis,^18^ among other biological functions, as well as their security when administered in humans in a phase I clinical trial.^19^ However, further research is still needed to establish pEVs in the clinical field.

Mass spectrometry (MS) is a powerful and valuable tool for characterizing the protein content of EVs, providing a key aspect in deciphering the biological role of EVs and exploring their potential use as diagnostic, screening, and therapeutic tool.^4,14^ Overall, the analysis of EVs using omics technologies involves the generation of massive data sets that can be compiled in public databases, helping to expand EV research and discovery.^21^ Advances in this technology has provided a considerable improvement in the understanding of EV components,^22^ especially in the identification of a large number of biomarkers associated with physiological and pathological mechanisms.^23^ However, the development of reliable and applicable EV-based biomarkers for clinical use remains a challenge, as even for the same disease, the results of EV analyses of patients’ blood are often inconsistent between studies. One of the main reasons behind these results is the variability in the experimental procedures.^24^ It is mandatory to direct studies toward the development of new techniques and the establishment of standardized protocols for sample processing, allowing to improve reproducibility and comparability.^1,7,18^ In this regard, a critical point is the utilization of an efficient method for EV cargo extraction, since it is essential that changes in the EV content profile accurately reflect biological conditions and are not influenced by technical limitations.^26^ Additionally, proper sample preparation prior to MS analysis is crucial, as chemical contaminants remaining from the preprocessing and isolation of EVs can be devastating.^27^ Thus, with the aim of contributing to the standardization of methodology in the field of pEV proteomics research, the present study provides a comparative analysis of different techniques for the extraction of pEV-derived proteins prior to MS analysis.

## Experimental Procedures

### Acquisition and Processing of PL

Human PL was obtained from the IdISBa Biobank with the approval of the Ethics Committee (IB1955/12 BIO ref 02/2021) upon ethical approval of the project by the CEI-IB (IB 4453/21 PI). To generate each platelet concentrate, a total of 50 buffy coats were used, rinsed with 0.9% NaCl, and centrifuged at 651*g* for 10 min. To generate PL, three freeze/thaw cycles (−80 °C/37 °C) and a freeze/thaw cycle (−80 °C/55 °C) were applied to platelet concentrates, followed by centrifugation at 5050*g* for 20 min and filtering via 40 μm porous membrane filters (Sartorius, Goettingen, Germany). The product was centrifuged at 1500*g* for 15 min at 4 °C, followed by centrifugation at 10,000*g* for 30 min at 4 °C. Supernatant was finally filtered through 0.8 and 0.2 μm porous membrane filters (Sartorius).

### pEV Isolation

Size exclusion chromatography (SEC) using the KTA go system (Cytiva, Marlborough, MA, USA) in combination with the HiPrep 26/60 Sephacryl 400HR column (AKTA) and the F9-R fraction collector (Cytiva) served to isolate pEV samples. A total of 3 different PLs (PL 1–3) were used for pEV isolation by injecting 10 mL into the system loop. Two different isolates were performed from PL 1, resulting in pEV of 1.1 and 1.2 samples. At a flow rate of 1.3 mL/min using Plasmalyte pH 7.4 (Viaflo, Italy) as the mobile phase, fractions 8, 9, and 10 were collected, pooled, and stored at −80 °C until analytical procedures.

### pEV Characterization

#### Nanoparticle Tracking Analysis

Size distribution and concentration of pEVs were analyzed using the Nanosight NS300 (Malvern Instruments, Malvern, UK), and data was processed with nanoparticle tracking analysis (NTA) 3.2 Dev Build 3.2.16 Software. Five different videos were obtained for each diluted sample (1/1000) with a camera level of 16, green laser type, and 25 frames per second.

#### Transmission Electron Microscopy

Visualization of the pEV morphology was achieved by transmission electron microscopy (TEM) (Talos F200i, Thermo Fisher Scientific, Waltham, MA, USA). Briefly, 4% formaldehyde (Sigma-Aldrich, Sant Louis, MO, USA) was added to samples in a 1:1 ratio, incubated on Formvar-Carbon coated grids (Ted Pella, CA, USA), and fixed with 1% glutaraldehyde (Sigma-Aldrich) for 5 min. After rinsing with deionized water, sample grids were stained with 2% tungstic acid (Electron Microscopy Sciences, Hatfield, PA, USA) and washed with deionized water. Images were taken at a current of 80 kV.

#### Western Blot

Detection of pEV-related protein biomarkers was performed by Western blotting (WB). Total protein content of the samples was measured spectrophotometrically (NanoDrop 2000/2000c, Thermo Fisher Scientific) or by Pierce BCA Protein Assay Kit (Thermo Fisher Scientific), following manufacturer’s instructions. CD9 and CD63 tetraspanins were detected by loading 2.5 μg of protein in a 12% polyacrylamide gel. Standard SDS-PAGE was performed, followed by humid transference to a nitrocellulose membrane (GE Healthcare, Pittsburgh, PA, USA). The blocking process required 10% dry skimmed milk (Central Lechera Asturiana, Asturias, Spain) in TBS with 10% Tween-20 (Scharlab, Barcelona, Spain) and consecutive overnight incubation at 4 °C with either antihuman CD9 monoclonal antibody (clone Ts9 diluted 1:2000, Thermo Fisher Scientific) or antihuman CD63 monoclonal antibody (clone TS63, diluted 1:5000, Abcam, Cambridge, UK). Thereafter, membranes were incubated for 1 h with HRP-coupled secondary antibody (diluted 1:2000, Thermo Fisher Scientific). Revealing process was conducted as follows. Membranes were submerged in Clarity Western ECL Substrate reagents (Bio-Rad, Hercules, CA, USA) and exposed to photosensitive films (GE Healthcare), which were subsequently immersed in revealing and fixation solutions (Carestream, Rochester, NY, USA).

HSC70 and albumin were detected after samples were lysed with RIPA 1× (Millipore, Burlington, MA, USA) and prepared with loading buffer under reducing environment (2.5% of β-mercaptoethanol). A total of 15 and 5 μg of protein from each sample were loaded onto 10% and 8% polyacrylamide gels, respectively. SDS-PAGE and humid transfer were performed in the same manner as described above. Blocking process was realized with TBS Odyssey Intercept Blocking Solution (LI-COR, Lincoln, USA), and membranes were incubated overnight at 4 °C with either antihuman HSC70 monoclonal antibody (clone B-6, diluted 1:1500, Santa Cruz, Dallas, TX, USA) or antihuman albumin monoclonal antibody (clone AL-01, diluted 1:2000, Invitrogen, Waltham, MA, USA). Finally, membranes were incubated with secondary antibody (IRDye 800CW Donkey anti-Mouse, LI-COR) diluted 1:8000 in the blocking solution TBS Odyssey with 0.1% Tween-20 for 1 h. Membranes revealing process was completed using the Odyssey Imaging System DiGit Blot scanner and Image Studio-Digits Software 4.0 (LI-COR).

### Protein Isolation and Digestion

#### Organic Solvent Precipitation

Extraction of protein and metabolites from pEVs samples was performed as follows. Absolute methanol was added to each sample in a 70:30 ratio and centrifuged at 18,213 rcf for 15 min at 4 °C. Supernatants containing metabolites were stored for future analysis, and the precipitates were resuspended with lysis buffer (50 mM Tris pH 7.4, 150 mM NaCl, 1 mM EDTA, 1% Triton X-100, protease inhibitor, phosphatase inhibitor and distilled water). Protein concentration was measured spectrophotometrically (NanoDrop 2000/2000c, Thermo Scientific). Pure isopropanol was added to 20 μg of protein from each sample, vortexed vigorously, and incubated at −20 °C overnight. The following day, samples were centrifuged at 18,213 rcf for 10 min at 4 °C. Supernatants were removed, and precipitates were allowed to dry on ice in a fume extraction hood. Pellets were resuspended in 50 mM ammonium bicarbonate (Scientific Laboratory Supplies, Dublin 9) and sonicated. Protein disulfide bridges were reduced by adding 1,4-dithiothreitol (Roche, Basel, Switzerland) for 30 min at 98 °C. Alkylation process involved the incorporation of 2-iodoacetamide (Sigma-Aldrich) and incubation of samples for 30 min at room temperature. Protein digestion was achieved by the addition of sequencing grade modified trypsin (Promega Biotech Ibérica, Madrid, Spain) and overnight standing of the samples at 37 °C. Finally, 2% trifluoroacetic acid (Sigma-Aldrich) in 20% acetonitrile (Thermo Fisher) was added to the digested proteins.

#### Sodium Dodecyl Sulfate Polyacrylamide Gel Electrophoresis

Standard SDS-PAGE methodology was performed. A 10% polyacrylamide gel was prepared, and 20 μg of protein was loaded after samples were concentrated with a centrifugal filtration system (Nanosep Centrifugal Devices with Omega Membrane, Cytiva) and lysed with lysis buffer (50 mM Tris pH 7.4, 150 mM NaCl, 1 mM EDTA, 1% Triton X-100, protease inhibitor, phosphatase inhibitor, and distilled water). 80 V for 30 min and 120 V for 10 min were programmed for short-term electrophoresis. Gels were stained following the Coomassie Blue staining protocol,^28^ and complete visible bands were cut out and transferred to deionized water. Cleanup and destaining of protein bands were performed by adding 400 mM ammonium bicarbonate (Scientific Laboratory Supplies) and acetonitrile (Thermo Fisher Scientific) and incubating the samples for 10 min with gentle agitation. This process was repeated thrice. Gels were allowed to dry at room temperature after a final rinsing step with 100 μL of acetonitrile. For on-gel protein digestion, sequencing grade modified trypsin (Promega Biotech Ibérica) diluted in ammonium bicarbonate (Scientific Laboratory Supplies) was included and stored overnight at 37 °C. Digested proteins were extracted from the gel as follows. A mix of acetonitrile 50% (Thermo Fisher Scientific) and trifluoroacetic acid 5% (Sigma-Aldrich) was added to samples and incubated under agitation for 10 min. Diluent was stored, and the process was repeated by adding acetonitrile (Thermo Fisher Scientific) to the gels. All collected extractions containing the digested proteins were pooled and stored until further analysis.

#### Magnetic Beads

Lysed samples from the SDS-PAGE methodology were used to initialize the magnetic bead-based protocol (Figure 1). Protein reduction was conducted by adding 10 mM 4-dithiothreitol (Roche) and incubating the samples for 30 min at 58 °C under agitation. Subsequent alkylation process involved the addition of 10 mM 2-iodoacetamide (Sigma-Aldrich) and the incubation of samples at room temperature for 30 min. Then, proteins were quenched with 10 mM 4-dithiothreitol (Roche). SpeedBeads magnetic carboxylate modified particles (Sigma-Aldrich) were conditioned according to manufacturer’s instructions. Stock beads were added to samples followed by the incorporation of absolute ethanol in 50:50 proportion. Samples were vortexed and incubated for 10 min at 25 °C and intense agitation. External magnetic force was applied and supernatants were removed. Magnetic beads (MB) were rinsed by adding 80% EtOH (Scharlab), vortexing, and centrifuging samples to remove supernatant. This step was repeated 3 times, and final precipitates were resuspended in 100 mM ammonium bicarbonate (Scientific Laboratory Supplies). Sequencing grade modified trypsin (Promega Biotech Ibérica) was added for protein digestion and incubated overnight at 37 °C under agitation. Digested proteins were obtained by using an external magnet and centrifugation process. Magnetic beads were additionally used for peptide purification along with the addition of pure acetonitrile (Thermo Fisher Scientific). Samples were incubated in agitation for 10 min at 25 °C. Using external magnetic force and centrifugation processes, supernatants were discarded, and beads were rinsed with acetonitrile (Thermo Fisher Scientific). Peptides were eluted with 2% dimethyl sulfoxide (DMSO) in combination with ultrasounds, centrifugation, and external magnet force.

**Figure 1:**
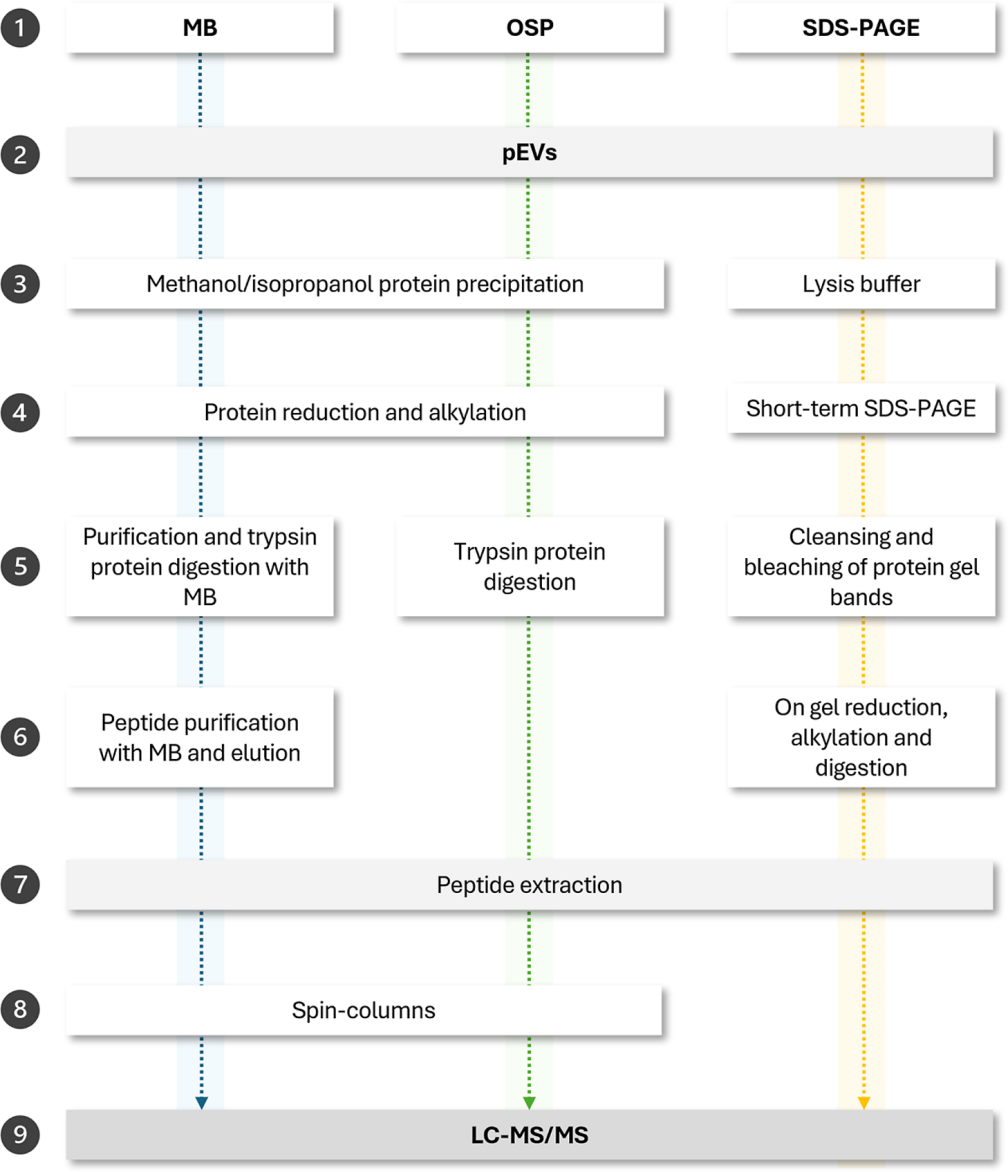
Graphical representation of the different methodologies used for protein extraction from pEV samples.

### Mass Spectrometry (Proteome) Analysis

All samples, including two technical duplicates for each, were loaded onto Pierce C18 Spin Columns (Thermo Fisher Scientific), following the manufacturer’s instructions, and concentrated using an integrated vacuum concentrator. Samples were resuspended in 20 μL H_2_O/formic acid 0.1% (FischerScientific, Hampton, NH, USA), and 1 μL was used for sample acquisition; peptides present in the sample were not quantified before acquisition.

All analyses were performed by a Q-Exactive-Orbitrap mass spectrometer (Thermo Fisher Scientific) equipped with a Nanospray Flex Ion Source and EASY-nLC 1200 peptide separation system (Thermo Fisher Scientific). For each sample, a total volume of 1 μL was loaded onto a PePMap NeO C18 precolumn (5 μm, 300 μm × 5 mm, 1500 bar) interfaced with a self-packed analytical column (DNV PePMap Neo C18, 2 μm, 75 μm × 500 mm, 100 Å, 1500 bar, Thermo Fisher Scientific), followed by a gradient elution of mobile phase (A: H_2_O 0.1% HCOOH and B: 80% CH_3_CN 0.1% HCOOH) with a constant flow of 300 nL/min (Figure S1). Complete MS scan acquisitions were performed over a 375–1500 *m*/*z* range with a 70,000 resolution.

Data-dependent mode was operated on the spectrometer, in which the 15 most abundant ions in each MS scan underwent MS/MS in the higher-energy collisional dissociation (HCD) cell under normalized collision energy of 28 and 17,500 resolution with dynamic exclusion of 40 s. Temperature of the ion transfer capillary was set at 275 °C, the atomization voltage was set at 1.9 kV in positive mode, and the radio frequency level of the S-lens was set at 50 AU, respectively.

Raw data files were processed using Proteome Discoverer 2.4.1.15 (Thermo Scientific). The LC–MS/MS data were searched against the UniProt protein database (February 2020, 20,303 entries), supplemented with a contaminant database (298 sequences). MS/MS spectra were searched with SequestHT search engine using the following settings: 10 ppm precursor mass tolerance and 0.1 Da fragment mass tolerance; up to 4 missed cleavages; minimum peptide length 6 amino acids; methionine oxidation (+15.995 Da), cysteine carbamidomethylation (+57.021 Da), acetyl (+42.01 Da, N termini), met-loss (−131.040 Da, N termini), and met-loss + acetyl (−89.030 Da, N termini) as dynamic modifications. The search engine results were then filtered for 1% false discovery rate (FDR) using the Percolator algorithm. At least two peptides were required for the protein identification. Only the areas of peptides that are not shared between different proteins or protein groups were used for the protein quantification.

### Statistics

Tests used for analysis of presented data include Shapiro–Wilk for normality test and student *t*-test or Mann–Whitney test for differences between groups with parametric and nonparametric distribution, respectively. GraphPad Prism 9 (La Jolla, CA, USA) software was used to perform all the analyses.

### Data Repository

The mass spectrometry proteomics data have been deposited to the figshare repository (http://figshare.com/) (10.6084/m9.figshare.25298638.v1) and to the ProteomeXchange Consortium via the PRIDE partner repository with the data set identifier PXD060357 and 10.6019/PXD060357.

### Bioinformatics

Biological functional analysis of resulting proteins was performed using FunRich software (v3.1.3), supported by the Vesiclepedia database (http://www.microvesicles.org/), accessed on (February 20, 2024). A comparative analysis was performed including three studies collected in the Vesiclepedia database under the identifiers: Vesiclepedia_406 (PubMed ID: 19806257),^29^ Vesiclepedia_520 (PubMed ID: 23601281),^30^ and Vesiclepedia_369 (PubMed ID: 16212402).^31^ These were selected based on the type of sample used (“platelets”), biomolecules identified (“proteins”), species (“”), and analysis (“proteomics”).

## Results

### pEV Isolation and Characterization

Isolation by SEC revealed enriched pEV fractions (8–10) identified by CD9 tetraspanin immunodetection (Figure S2). All samples reached a concentration order of 10^11^ particles per milliliter (Table 1) with significant differences between the different isolates, each from a different platelet lysate obtained from a pool of 50 donors (*p* < 0.05).

**Table 1:** pEV Characterization as a Function of Particle Size, Concentration, Protein Content, and Purity of the Samples^a^

	pEV 1	pEV 2	pEV 3
pEV’s size, nm (mean ± SEM)	116 ± 3	127 ± 2^a^	123 ± 1
pEV’s concentration, part/mL (mean ± SEM)	1.83 × 10^11^ ± 7.69 × 10^9^	1.38 × 10^11^ ± 8.16 × 10^9a^	1.90 × 10^11^ ± 3.57 × 10^9b^
protein concentration, μg/μL	0.295	0.166	0.119
purity (particles/μg)	6.20 × 10^8^	8.31 × 10^8^	1.60 × 10^9^

a pEV 1, pEV 2, and pEV 3 constitute three biological replicates, each obtained from a different platelet lysate pool of 50 donors. Data from particle size and concentration were statistically compared using an independent samples *t*-test ((^a^ *p* < 0.05 vs pEV 1, ^b^ *p* < 0.05) vs pEV 2).

Morphology of pEVs evaluated by TEM (Figures 2A and S3) was consistent with particle size results obtained by NTA, with statistical differences (*p* < 0.05) among pEV 1 and pEV 2 samples. Enrichment in pEV was demonstrated by an increased band intensity of CD9 and CD63 tetraspanins compared to the original LPs, when detected by Western blot. Similarly, pEV samples were positive for the cytosolic marker HSC70, showing a similar pattern for all samples. Additionally, coisolated albumin was considerably decreased in pooled fractions of pEVs (Figures 2B and S4), evidencing the correct pEV enrichment process by means of SEC.

**Figure 2:**
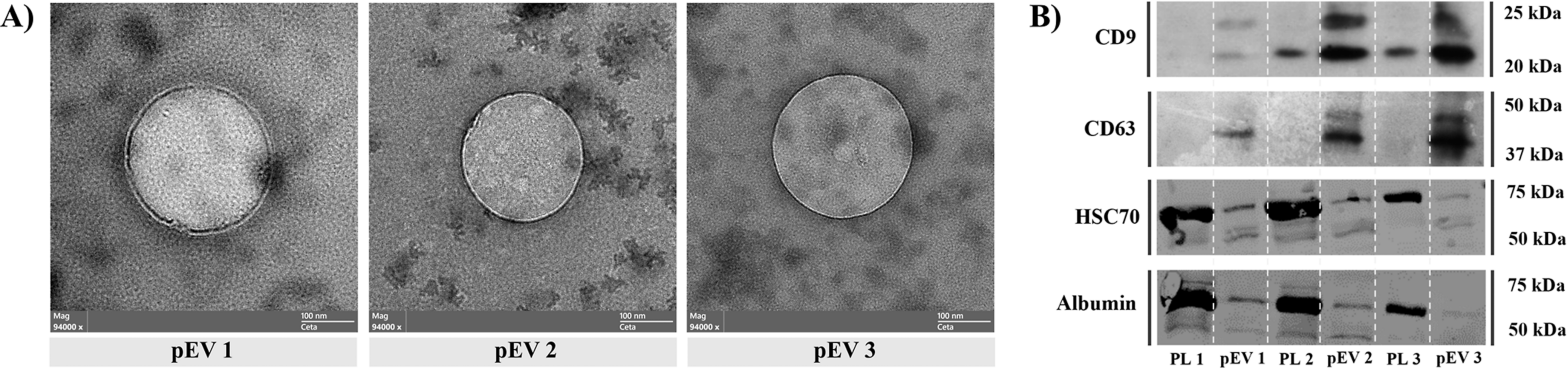
pEV’s characterization by Western blot and TEM. (A) Representative images obtained by transmission electron microscopy (TEM) for pEV samples acquired at 94,000× magnification and 80 kV. (B) Identification of characteristic markers of EVs by Western blot. Tetraspanins CD9 and CD63, cytosolic HSC70, and coisolated albumin were identified in the different pEV samples, as well as in their respective original samples (PL). pEV1, pEV2, and pEV3 constitute three biological replicates, each obtained from a different platelet lysate pool of 50 donors.

### Proteomic Analysis and Bioinformatics

Principal component analysis (PCA) evidenced a clustering of the samples according to the performed protein extraction methodology, as shown in Figure 3A. Similarly, technical duplicates of samples appeared close together in the plot, except for one pEV 1.1 duplicate, regarding MB methodology, which was considered outlier and excluded from analysis (data not shown). To perform an additional control quality assay, one technical duplicate from pEV 1.1 and two technical duplicates from pEV 1.2 samples, from MB, were acquired twice in LC–MS/MS (Table S1). Notwithstanding the grouping of samples, normalized abundances of identified proteins acquired similar patterns when protein extraction methodologies were compared since no statistically significant differences were found between groups (Figure 3B). Equally, a total of 275 proteins were identified (258 using MB, 269 using SDS-PAGE, and 268 using OSP), revealing a reduced number of unique proteins per methodology and a value of 91.3% of commonly distributed proteins observed by all three procedures (Figure 3C).

**Figure 3:**
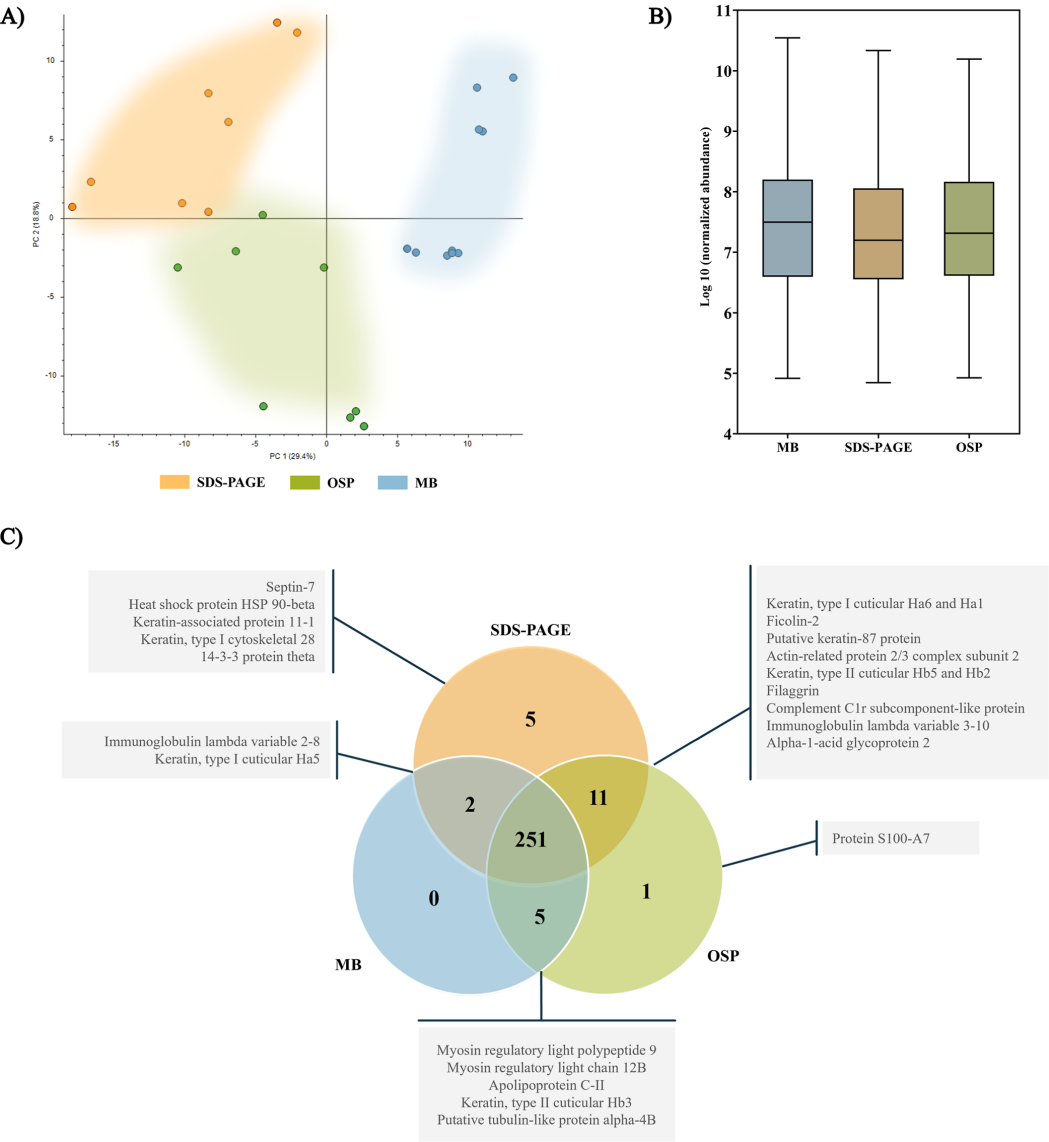
Results of the proteomic analysis related to the comparative methodology. (A) Principal component analysis (PCA) applied to the data set. The clustering areas associated with the SDS-PAGE, OSP, and MB methodologies are represented in red, green, and blue, respectively. (B) Normalized protein abundances. Diagram was generated by calculating the arithmetic means of the normalized abundances of each protein detected in the different samples, considering each methodology. Results were statistically compared with Kruskal–Wallis’ test. (C) Venn diagram depicting the number of common and unique proteins related to each methodology.

Resulting protein profiles (Tables S2–S4) were analyzed using Vesiclepedia database through FunRich 3.1.3 software using associated genes as data input. Four common immunoglobulin-related proteins (Immunoglobulin mu heavy chain, Immunoglobulin alpha-2 heavy chain, Immunoglobulin kappa light chain, and Immunoglobulin lambda-1 light chain) were excluded from the analysis as no recognized genes were associated. Concerning the total number of proteins in the data set, a total of 250, 252, and 243 proteins associated with SDS-PAGE, OSP, and MB, respectively, were present in the Vesiclepedia database (Figure 4A). Similarly, the protein extraction methods analyzed allow the detection of proteins classified as Top 100 EV markers in the Vesiclepedia Database (Figure 4B).

**Figure 4:**
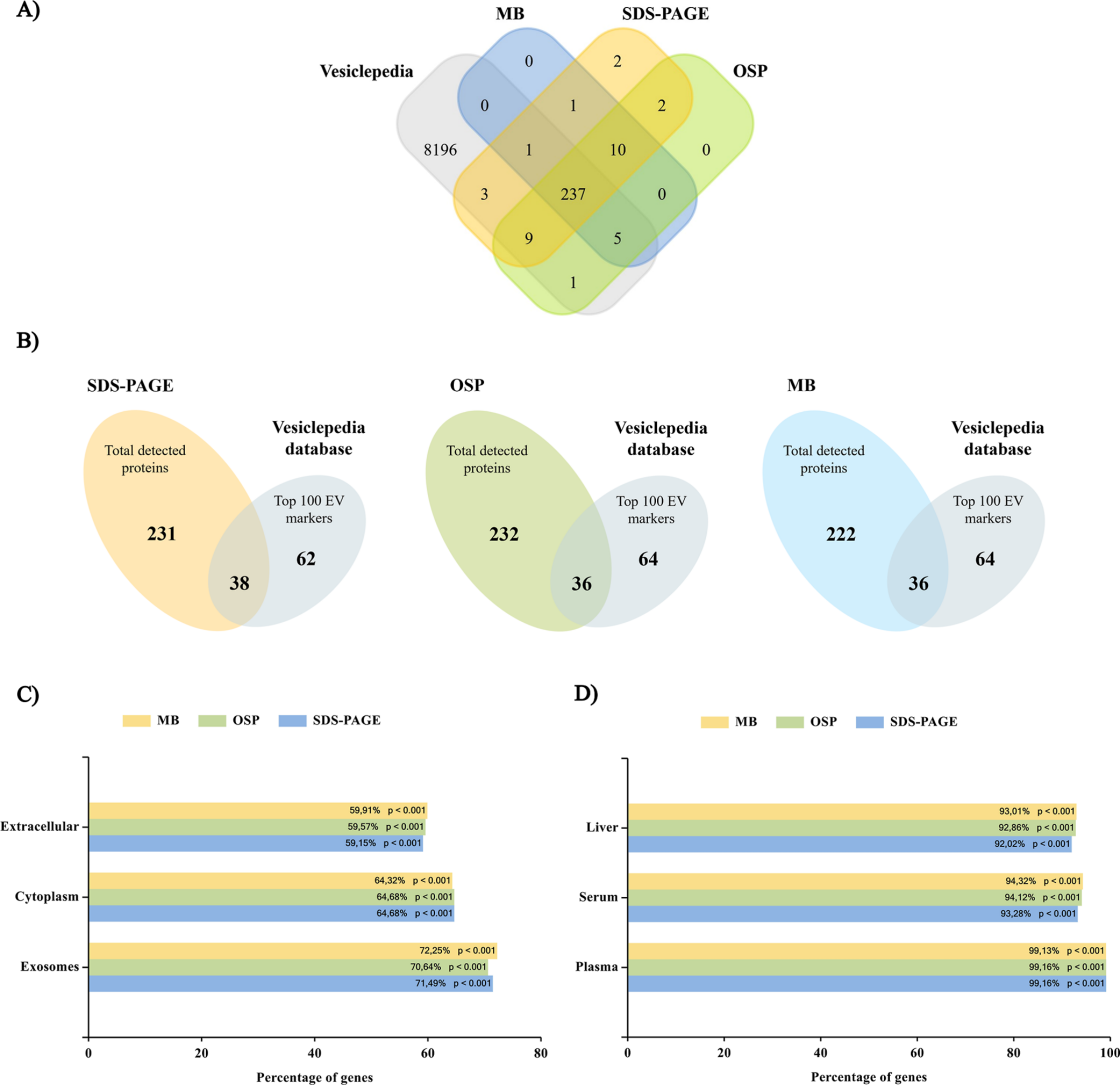
Bioinformatic analysis of protein data set. (A) Venn diagram representing the number of proteins identified under each methodology and listed in the Vesiclepedia database under “human” and “protein” terms. (B) Common proteins identified under each methodology and listed in the Vesiclepedia database Top 100 EV markers. Enrichment analysis of (C) cellular components and (D) site of expression categories using FunRich database as background.

Functional enrichment analysis revealed exosome, cytoplasm, and extracellular environment as the most significant findings for cellular component category (Figure 4C) and plasma, serum, and liver as the main expression site-associated outputs (Figure 4D). As for both cases, percentage of genes involved in each category and the significance value were strongly comparable across methodologies.

To compare the proteomic profile of our study, we included published data from three studies reported in the Vesiclepedia database^29–31^ in which the proteome of pEVs had been analyzed. The SDS-PAGE methodology revealed 138 common proteins with any of the three studies mentioned above, of which 38 correspond to proteins included in the top 100 EV markers described in the Vesiclepedia database. The MB and OSP methodologies had 137 proteins in common with the other studies, of which 36 correspond to proteins included in the top 100 EV markers described in the Vesiclepedia database (Table S5). If we restricted the comparison criteria to common proteins in the three included studies together with our proteomic profiling, we found only 36 proteins for the SDS-PAGE and MB methodologies and 37 proteins for the OSP methodology (Table 2). Among this list, multiple proteins are considered EV markers and are included in the top 100 EV markers of the Vesiclepedia database.

**Table 2:** Comparative Analysis of Common Proteins Identified in Other Proteomics Studies of Platelet-Derived Extracellular Vesicles^a^

SDS-PAGE	OSP	MB	protein recommended name
X	X	X	**Alpha-actinin-1**
X	X	X	**Albumin**
X	X	X	Apolipoprotein B-100
X	X	X	**Complement C3**
X	X	X	**Adenylyl** **cyclase-associated** **protein 1**
X	X	X	Caveolae Associated Protein 2
X	X	X	**CD9 antigen**
X	X	X	**Chloride intracellular channel** **protein 1**
X	X	X	**Alpha-enolase**
X	X	X	Fermitin family homologue 3
X	X	X	Fibrinogen alpha chain
X	X	X	Fibrinogen beta chain
X	X	X	Fibrinogen gamma chain
	X		Filaggrin-2
X	X	X	**Glyceraldehyde-3-Phosphate** **Dehydrogenase**
X	X	X	Platelet glycoprotein Ib beta chain
X	X	X	**Gelsolin**
X	X		Hemoglobin Subunit Beta
X	X	X	**Heat shock cognate** **71 kDa** **protein**
X	X	X	Integrin alpha-IIb
X	X	X	Integrin beta-3
X	X	X	LIM and senescent cell antigen-like-containing domain protein 1
X	X	X	Myosin light polypeptide 6
	X	X	Myosin regulatory light polypeptide 9
X	X	X	Beta-parvin
X	X	X	**Profilin-1**
X	X	X	**Pyruvate kinase PKM**
X	X	X	Pleckstrin
X	X	X	**Peptidyl-prolyl cis–trans** **isomerase A**
X	X	X	Stomatin
X	X	X	Serotransferrin
X	X	X	Thrombospondin-1
X	X	X	**Talin-1**
X			Tubulin alpha-4A chain
		X	Tubulin beta-1 chain
X	X	X	**Vinculin**
X	X	X	WD repeat-containing protein 1
X	X	X	**14-3-3 protein** **beta/alpha**
X	X	X	**14-3-3 protein** **zeta/delta**

a Note: protein names in **bold** are included in the top 100 EV markers according to the Vesiclepedia database. The name of the proteins has been selected based on the name recommended by UniProtKB/Swiss-Prot consulted through the GeneCards human gene database.

Among these unique proteins, the methodologies differ from each other minimally in the proteins exclusively identified, being Filaggrin, Keratin 28, and Keratin Associated Protein 11 for SDS-PAGE; Keratin 6B and S100-A7 for the OSP; and Keratin 6A for the MB (Table S6).

## Discussion

Research interest in EVs has increased in recent decades due to their multiple characteristics and their complex role in cellular communication based on their protected biological cargo.^1^ Because the biomolecular profile of EVs depends on their source and function (“*unde venis et quo vadis*”), analyzing their content serves as a powerful means of gaining valuable knowledge.^3^ To this end, MS is a crucial technology for deciphering their content and gaining the knowledge needed to apply EVs as diagnostic or therapeutic tools.^20^ However, reproducibility and performance concerns associated with proteomics methods for plasma derived EVs remain evident,^32^ which diminishes their transfer to clinical practice.^25^ In this context, this study aims to contribute to the standardization of protocols used in the analysis of pEV content by comparing three methodologies for protein extraction prior to LC–MS/MS analysis.

Three different platelet lysates (PL 1–3) were used to isolate pEV samples by SEC, obtaining one pool of pEV-enriched fractions from each one, with the exception of PL 1, of which two different isolates were obtained (pEV 1.1 and pEV 1.2). Platelet concentrates such as PL have become of great interest for their applications in regenerative medicine or tissue engineering, among others, and their functional effects have been standardized by analyzing their proteomic profile.^33^ Although various protocols for generating PL can be found in the literature,^34^ a critical aspect of the process is the activation of platelets, which promotes the release of bioactive substances^35^ and pEVs.^33^ In this regard, freeze–thawing has proven to be the most widely used technique in the LP generation process,^34^ which makes LP production relatively simple and applicable in the hospital environment.^36^

Although this study does not address a comparison of EV isolation methods, previous studies proposed SEC as an optimal tool for this purpose^32^ and especially for the isolation of pEVs from the different proteins present in the PL.^33,37^ One of its main advantages is the preservation of the biological activity of the EVs^22^ and the higher plasma EV recovery, despite none of the actual existing methods leading to pure preparations.^38^ Characterization of the obtained pEVs reflected significant differences (*p* < 0.05) between samples in terms of concentration, as well as in the average particle size, whereas all of them had a concentration order of 10 raised to 11 and a mean size range of 115–130 nm. The purity of the samples revealed variability between the different pEV isolates. The limit of 1.5 × 10^9^ particles/μg is classified as impure according to Webber and Clayton, 2013,^39^ when calculating the ratio of particle to protein concentration. It should be noted that this classification was based on cell culture media or urine-derived EV, which tend to have a lower protein content compared to those derived from plasma.^40^ The definition of quality standards in the field of EVs is a current challenge, as the establishment of thresholds depends on the source of EVs, the isolation method, and the final application.^41^ While it has been established that a high purity EV sample should be equal to or greater than 3 × 10^10^ particles/μg protein, complex biological fluids, such as plasma, significantly increase the difficulty in achieving this purity.^39^

Concerning marker characterization, all samples exhibited an enrichment in the CD9 and CD63 tetraspanins, together with a reduction in the intensity of bands for the cytosolic marker HSC70 and for albumin as coisolation contaminant, complying with the specifications of the International Society for Extracellular Vesicles.^42^

Overall, the different methodologies analyzed revealed a total of 275 identified proteins, 91.3% of which were common to all three procedures. Although samples exhibited a clear association in the PCA graph according to the methodology involved, the mean normalized abundances for the identified proteins did not show statistically significant differences between methodologies, which correlates with the minimal number of uniquely identified proteins per methodology (5, 1 and 0, for SDS-PAGE, OSP and MB, respectively). Protein S100-A7, a multifunctional protein with antimicrobial activity in keratinocytes,^43^ was only detected in the OSP methodology. The SDS-PAGE methodology identified keratins (Keratin-associated protein 11-1 and Keratin, type I cytoskeletal 28), septin 7 protein, Heat shock protein HSP 90-beta, and 14-3-3 protein theta, considering that the last three are present in platelets.^44–46^ This minimal difference in uniquely identified proteins, when both the results of the proteins identified in our study and data from other publications, shows the robustness of the three methodologies to be used for protein extraction from pEVs. Notably, keratin-related proteins, often identified as common contaminants in MS analysis, remain challenging to exclude due to the difficulty in limiting sample exposure to this protein.^47^ These results are consistent with previous studies that have determined similar values for the total number of proteins identified when analyzing samples of human platelet lysates^48^ or platelet-derived EV subpopulations^49^ by mass spectrometry. However, a major consideration is the presence of protein contaminants from the biological fluid of the origin of the EVs. After digestion of the samples, a high number of contaminant proteins involves a masking of low-abundance peptides produced by EVs,^50^ which reduces the total number of proteins detected.^34^ Despite the advantages of SEC for EV isolation, the isolates maintained residual lipoprotein contamination. Whereas this could be improved by combining SEC with other methodologies, the minimal presence of contaminants such as albumin particles would remain.^51^ Although albumin is found among the proteins detected, our results show a considerable reduction of this protein in the pEV samples in comparison with the source samples after isolation by SEC.

Bioinformatic analysis of the resulting protein list demonstrates strongly similar patterns to the enrichment analysis. The three methodologies provide a set of proteins that reveal exosomes, cytoplasm, and cellular environment as the main results associated with the cellular component, with a very close percentage of genes between methodological groups. Similarly, the enrichment analysis results for expression site highlights plasma, serum, and liver as the most prominent results. These data indicate a strong correlation between the proteins identified for each approach, making evident the similarity that exists in terms of reproducibility and performance for the three methodologies analyzed. This fact has been reported previously in the literature, highlighting that the number of proteins identified by MS does not depend on the type of sample or the methodology used in its preparation.^27^ Moreover, the large number of matching proteins already collected in the Vesiclepedia database also contributes to verifying the correct isolation of pEV and their protein content. Despite the similarity in obtained results, the intrinsic characteristics of each methodology (Table 3) must be considered. First, after the protein extraction process, different amounts of peptides are obtained depending on the methodology used, with SDS-PAGE yielding the highest number of peptides and MB yielding the lowest (Table S1). Second, proteomic analysis commonly involves a first step using lysis buffer with or without detergent for protein extraction,^52^ with exception of the currently described organic solvent-based protein extraction methodology, which replaces this lysis process by the separation of biological components in two phases. This methodology involves commonly used reagents and minimal working time with the main advantage of obtaining proteins and metabolites from a unique sample. In fact, the use of absolute methanol as an optimal method to clearly separate between proteins and an adequate metabolite profile has been previously described.^53^ However, this fact also contributes to interphase contamination and lower specificity to the process. In turn, electrophoresis gel is an effective and simple strategy to remove potential contaminants based on the molecular weight of proteins, benefiting the ulterior MS analysis.^52^ However, the on-gel digestion complicates the digestion process due to the presence of proteins in the gel matrix and reduces the total amount of peptides recovered,^54^ comprising a long-term work time. Finally, magnetic particles stand out as a new tool for the detection of peptides and proteins, since their small size in combination with their high binding capacity makes them a highly sensitive method for application to MS.^55^ Despite its advantages, the cost of the technique is significantly higher than those of the other two methods.

**Table 3:** Summary of Different Protein Isolation Methodologies^a^

	OSP	SDS-PAGE	MB
principle	protein precipitation by organic solvents	protein migration by short SDS-electrophoresis	immunocapture of proteins and peptides by magnetic particles
pros	commonly used reagents	short standard SDS-PAGE	high binding capacity
	additional metabolite obtention	remove potential contaminants	dual detection of proteins and peptides
cons	lower specificity	reduced on-gel digestion	increased cost
	interphase biomolecule mixing	less recovered peptides	
		limited sample quantity	
time consuming	*	***	**

a Note: time consuming is indicated by (*), with a higher number of these symbols correlating with a longer working time.

While protein extraction is a critical step in mass spectrometry analysis, new technologies applied for biomarker discovery should also be considered. Compared to data dependent acquisition (DDA) methodologies, data independent acquisition (DIA) approach presents advantages in terms of sensitivity, reproducibility, and detection range.^56^ Hence, recent studies have obtained promising results in the field of circulating-EV biomarkers using new DIA strategies such as sequential window acquisition of all theoretical mass spectra (SWATH)-DIA^57^ or 4D-data-DIA.^58^

The main limitations of this study are the limited number of samples, the exclusive focus on a single source of origin, and the use of only one pEV isolation technique. Additionally, the variations in protein yield between the extraction methods should be carefully considered when selecting the most appropriate technique. Another limitation of the study is the variability of peptides obtained after each methodology. Even though for LC–MS/MS injection, samples were equalized by volume and not by peptide amount, results were similar for the three processes. Nonetheless, these protein extraction techniques are expected to be applicable to other EV samples derived from biological fluids and isolated using SEC.

In conclusion, mass spectrometry represents a valuable tool for the characterization of protein-associated EVs. Although numerous studies have evaluated the optimal EV isolation technique for downstream MS analysis, the literature on EV protein isolation techniques is limited. Hence, we present three pEV-related protein extraction methodologies that are strongly similar in terms of yield and could be considered in attending to the research requirements and the intrinsic characteristics of each protocol. Further research will be needed to standardize protocols related to sample preprocessing prior to MS, contributing to the translation of pEVs into clinical practice as a potential therapeutic and diagnostic tool.

## Supplementary Material



## Data Availability

We have submitted all relevant data of our experiments to the EV-TRACK knowledge (EV-TRACK ID: EV240022)^59^ and to the figshare repository (http://figshare.com/) (10.6084/m9.figshare.25298638.v1).

## Notes

Gradual elution of mobile phase (B: 80% CH_3_CN 0.1% HCOOH) into the mass spectrometer; characterization of CD9 tetraspanin in fractions (2–14) obtained by SEC using Plasmalyte as eluent and hPL as sample; additional images obtained by TEM for pEV samples acquired at 94,000× magnification; Western blot images of the entire membranes derived from the characterization of CD9, CD63, HSC70 of pEV samples, as well as their respective original samples (PL); analyzed samples per methodology and total runs performed; protein profile obtained for SDS-PAGE; protein profile obtained for OSP; protein profile obtained for MB; associated gene names of common proteins identified in other published studies; and associated gene names of unique proteins only identified in our study (PDF)
