# Supplementary material for: Comparative Analysis of Platelet-Derived Extracellular Vesicle Protein Extraction Methodologies for Mass Spectrometry
Source: J Proteome Res. 2025 Jul 21;24(8):3931–42. doi: 10.1021/acs.jproteome.5c00089 (PMC12323001; doi:10.1021/acs.jproteome.5c00089)
Supplement: Supplementary file 1 [file pr5c00089_si_001.pdf]

Supporting Information

Comparative analysis of platelet-derived extracellular vesicles protein extraction methodologies for mass spectrometry

Carmen Ráez-Meseguer[1,2,3], Andreu Miquel Amengual-Tugores[1,2,3], Maria Antònia Forteza-Genestra[1,2,3], Francisca Orvay-Pintos[4], Rosa M. Gomila[4], Gabriel Martorell-Crespí[4], Javier Calvo[1,2,5], Antoni Gayà[1,2,5], Marta Monjo[1,2,3] y Joana Maria Ramis[1,2,3*]*

[1]Group of Cell Therapy and Tissue Engineering (TERCIT), Research Institute on Health Sciences (IUNICS), University of the Balearic Islands (UIB), Palma, 07122, Spain. [2]Health Research Institute of the Balearic Islands (IdISBa), Palma, 07120, Spain. [3]Department of Fundamental Biology and Health Sciences, UIB, Palma 07122, Spain. [4]Scientific Technical Service (SCT), UIB, Palma, 07122, Spain. [5]Fundació Banc de Sang i Teixits de les Illes Balears (FBSTIB), Palma, 07004, Spain.

Corresponding author: Marta Monjo (marta.monjo@uib.es); Joana Maria Ramis (joana.ramis@uib.es).

Table of Contents

Figure S1. Gradual elution of mobile phase (B: 80 % CH_3CN 0.1 % HCOOH) into the mass spectrometer.
Figure S2. Characterization of CD9 tetraspanin in fractions (2-14) obtained by SEC using Plasmalyte as eluent and hPL as sample.
Figure S3. Additional images obtained by TEM for pEV samples acquired at 94 000 x magnification.
Figure S4. Western blot images of the entire membranes derived from the characterization of CD9, CD63, HSC70 of pEV samples, as well as their respective original samples (PL).
Table S1. Analyzed samples per methodology and total runs performed
Table S2. Protein profile obtained for SDS-PAGE.
Table S3. Protein profile obtained for OSP.
Table S4. Protein profile obtained for MB.
Table S5. Associated gene names of common proteins identified in other published studies.
Table S6. Associated gene names of unique proteins only identified in our study.

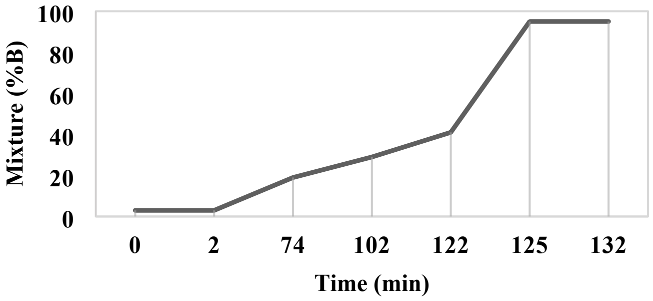

Supplementary Figure 1. Gradual elution of mobile phase (B: 80 % CH3CN 0.1 % HCOOH) into the mass spectrometer.

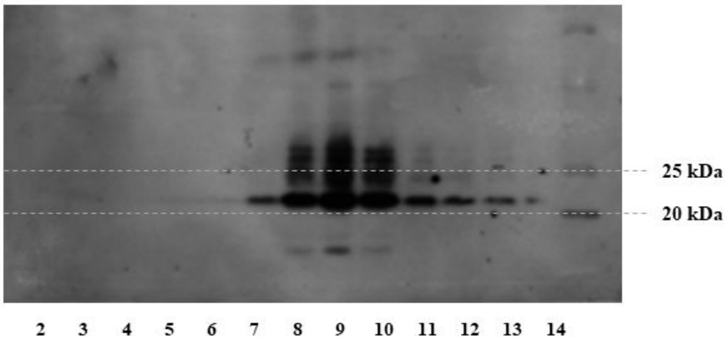

Supplementary Figure 2. Characterization of CD9 tetraspanin in fractions (2-14) obtained by SEC using Plasmalyte as eluent and hPL as sample.

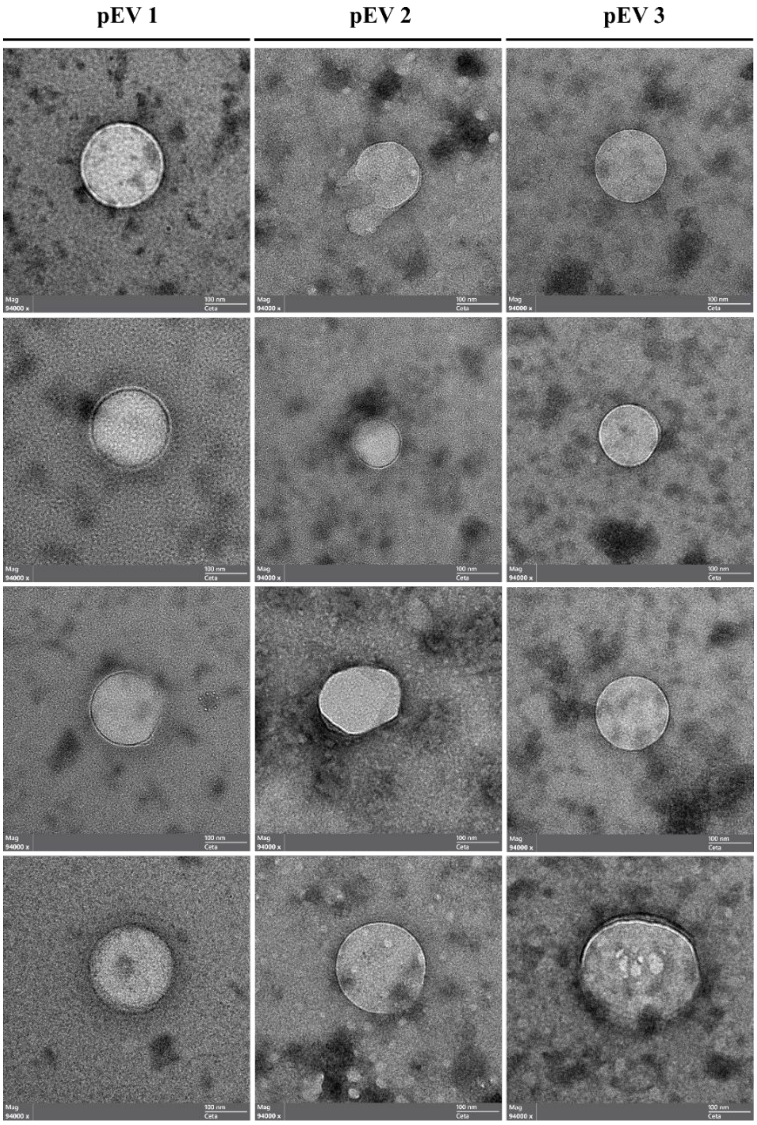

Supplementary Figure 3. Additional images obtained by TEM for pEV samples acquired at 94 000 x magnification.

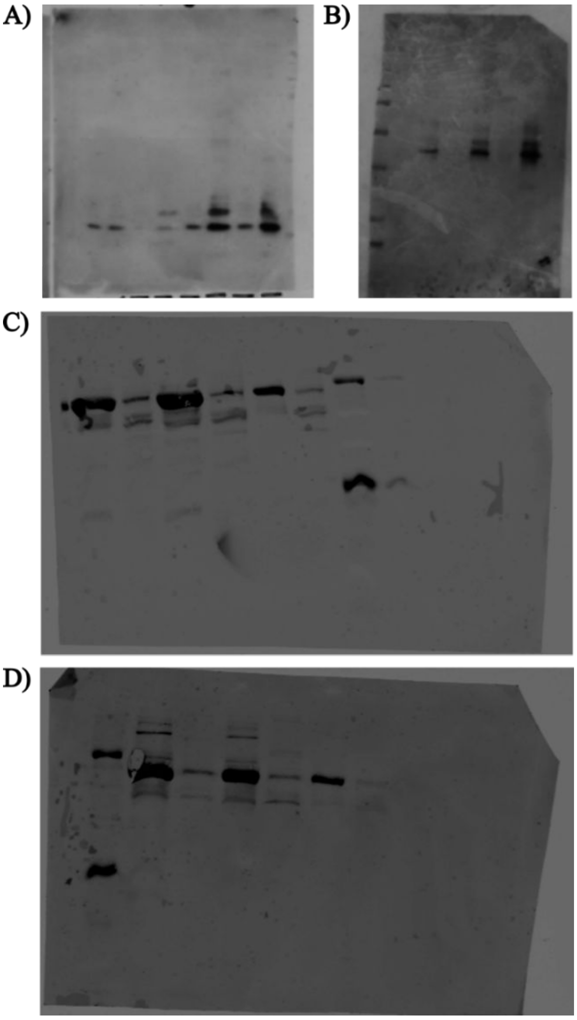

Supplementary Figure 4. Western blot images of the entire membranes derived from the characterization of CD9, CD63, HSC70 of pEV samples, as well as their respective original samples (PL).

Supplementary Table 1. Analyzed samples per methodology and total runs performed.

	Samples analysed according to methodology	Total runs per sample	Protein concentration prior to LC/MS-MS acquisition (mean ± SEM)
**SDS-PAGE**	pEV 1.1, pEV 1.2, pEV 2 and pEV 3	2	0,375 ± 0,026
**OSP**	pEV 1.1, pEV 1.2, pEV 2 and pEV 3	2	0,164 ± 0,026
**MB**	pEV 1.1†, pEV 1.2*, pEV 2 and pEV 3	2<br>2†<br>4*	0,032 ± 0,006

†One technical replicate of this sample was considered an outlier and was not considered for further analysis. *For this sample, 4 total runs were performed as an additional quality control.

Supplementary Table 2. Protein profile obtained for SDS-PAGE.

Accesion number	Description
P60709	Actin, cytoplasmic 1 OS=Homo sapiens OX=9606 GN=ACTB PE=1 SV=1
P07437	Tubulin beta chain OS=Homo sapiens OX=9606 GN=TUBB PE=1 SV=2
P68133	Actin, alpha skeletal muscle OS=Homo sapiens OX=9606 GN=ACTA1 PE=1 SV=1
P01834	Immunoglobulin kappa constant OS=Homo sapiens OX=9606 GN=IGKC PE=1 SV=2
P04264	Keratin, type II cytoskeletal 1 OS=Homo sapiens OX=9606 GN=KRT1 PE=1 SV=6
P35527	Keratin, type I cytoskeletal 9 OS=Homo sapiens OX=9606 GN=KRT9 PE=1 SV=3
P02768	Serum albumin OS=Homo sapiens OX=9606 GN=ALB PE=1 SV=2
P68366	Tubulin alpha-4A chain OS=Homo sapiens OX=9606 GN=TUBA4A PE=1 SV=1
Q9HBI1	Beta-parvin OS=Homo sapiens OX=9606 GN=PARVB PE=1 SV=1
P01857	Immunoglobulin heavy constant gamma 1 OS=Homo sapiens OX=9606 GN=IGHG1 PE=1 SV=1
P35908	Keratin, type II cytoskeletal 2 epidermal OS=Homo sapiens OX=9606 GN=KRT2 PE=1 SV=2
P14618	Pyruvate kinase PKM OS=Homo sapiens OX=9606 GN=PKM PE=1 SV=4
P01008	Antithrombin-III OS=Homo sapiens OX=9606 GN=SERPINC1 PE=1 SV=1
P00450	Ceruloplasmin OS=Homo sapiens OX=9606 GN=CP PE=1 SV=1
P04406	Glyceraldehyde-3-phosphate dehydrogenase OS=Homo sapiens OX=9606 GN=GAPDH PE=1 SV=3
P13645	Keratin, type I cytoskeletal 10 OS=Homo sapiens OX=9606 GN=KRT10 PE=1 SV=6
P27918	Properdin OS=Homo sapiens OX=9606 GN=CFP PE=1 SV=2
P04217	Alpha-1B-glycoprotein OS=Homo sapiens OX=9606 GN=A1BG PE=1 SV=4
P27169	Serum paraoxonase/arylesterase 1 OS=Homo sapiens OX=9606 GN=PON1 PE=1 SV=3
P13647	Keratin, type II cytoskeletal 5 OS=Homo sapiens OX=9606 GN=KRT5 PE=1 SV=3
Q9H4B7	Tubulin beta-1 chain OS=Homo sapiens OX=9606 GN=TUBB1 PE=1 SV=1
Q96PD5	N-acetylmuramoyl-L-alanine amidase OS=Homo sapiens OX=9606 GN=PGLYRP2 PE=1 SV=1
P00558	Phosphoglycerate kinase 1 OS=Homo sapiens OX=9606 GN=PGK1 PE=1 SV=3
P68363	Tubulin alpha-1B chain OS=Homo sapiens OX=9606 GN=TUBA1B PE=1 SV=1
Q96IY4	Carboxypeptidase B2 OS=Homo sapiens OX=9606 GN=CPB2 PE=1 SV=2
P00734	Prothrombin OS=Homo sapiens OX=9606 GN=F2 PE=1 SV=2
Q9Y490	Talin-1 OS=Homo sapiens OX=9606 GN=TLN1 PE=1 SV=3
P08603	Complement factor H OS=Homo sapiens OX=9606 GN=CFH PE=1 SV=4
P07360	Complement component C8 gamma chain OS=Homo sapiens OX=9606 GN=C8G PE=1 SV=3
Q562R1	Beta-actin-like protein 2 OS=Homo sapiens OX=9606 GN=ACTBL2 PE=1 SV=2
P52907	F-actin-capping protein subunit alpha-1 OS=Homo sapiens OX=9606 GN=CAPZA1 PE=1 SV=3
P07225	Vitamin K-dependent protein S OS=Homo sapiens OX=9606 GN=PROS1 PE=1 SV=1
P02675	Fibrinogen beta chain OS=Homo sapiens OX=9606 GN=FGB PE=1 SV=2
P30101	Protein disulfide-isomerase A3 OS=Homo sapiens OX=9606 GN=PDIA3 PE=1 SV=4
P06396	Gelsolin OS=Homo sapiens OX=9606 GN=GSN PE=1 SV=1
P01031	Complement C5 OS=Homo sapiens OX=9606 GN=C5 PE=1 SV=4
P02790	Hemopexin OS=Homo sapiens OX=9606 GN=HPX PE=1 SV=2

P08514	Integrin alpha-IIb OS=Homo sapiens OX=9606 GN=ITGA2B PE=1 SV=3
Q14624	Inter-alpha-trypsin inhibitor heavy chain H4 OS=Homo sapiens OX=9606 GN=ITIH4 PE=1 SV=4
P02760	Protein AMBP OS=Homo sapiens OX=9606 GN=AMBP PE=1 SV=1
P48740	Mannan-binding lectin serine protease 1 OS=Homo sapiens OX=9606 GN=MASP1 PE=1 SV=3
P01009	Alpha-1-antitrypsin OS=Homo sapiens OX=9606 GN=SERPINA1 PE=1 SV=3
P01859	Immunoglobulin heavy constant gamma 2 OS=Homo sapiens OX=9606 GN=IGHG2 PE=1 SV=2
P00738	Haptoglobin OS=Homo sapiens OX=9606 GN=HP PE=1 SV=1
P35858	Insulin-like growth factor-binding protein complex acid labile subunit OS=Homo sapiens OX=9606 GN=IGFALS PE=1 SV=1
P40197	Platelet glycoprotein V OS=Homo sapiens OX=9606 GN=GP5 PE=1 SV=1
P08567	Pleckstrin OS=Homo sapiens OX=9606 GN=PLEK PE=1 SV=3
O75083	WD repeat-containing protein 1 OS=Homo sapiens OX=9606 GN=WDR1 PE=1 SV=4
P01023	Alpha-2-macroglobulin OS=Homo sapiens OX=9606 GN=A2M PE=1 SV=3
Q9ULV4	Coronin-1C OS=Homo sapiens OX=9606 GN=CORO1C PE=1 SV=1
Q06033	Inter-alpha-trypsin inhibitor heavy chain H3 OS=Homo sapiens OX=9606 GN=ITIH3 PE=1 SV=2
P04004	Vitronectin OS=Homo sapiens OX=9606 GN=VTN PE=1 SV=1
P02746	Complement C1q subcomponent subunit B OS=Homo sapiens OX=9606 GN=C1QB PE=1 SV=3
P51884	Lumican OS=Homo sapiens OX=9606 GN=LUM PE=1 SV=2
P07358	Complement component C8 beta chain OS=Homo sapiens OX=9606 GN=C8B PE=1 SV=3
P02749	Beta-2-glycoprotein 1 OS=Homo sapiens OX=9606 GN=APOH PE=1 SV=3
P02774	Vitamin D-binding protein OS=Homo sapiens OX=9606 GN=GC PE=1 SV=2
Q86UX7	Fermitin family homolog 3 OS=Homo sapiens OX=9606 GN=FERMT3 PE=1 SV=1
P00736	Complement C1r subcomponent OS=Homo sapiens OX=9606 GN=C1R PE=1 SV=2
P21333	Filamin-A OS=Homo sapiens OX=9606 GN=FLNA PE=1 SV=4
P01871	Immunoglobulin heavy constant mu OS=Homo sapiens OX=9606 GN=IGHM PE=1 SV=4
P00742	Coagulation factor X OS=Homo sapiens OX=9606 GN=F10 PE=1 SV=2
P09871	Complement C1s subcomponent OS=Homo sapiens OX=9606 GN=C1S PE=1 SV=1
P35579	Myosin-9 OS=Homo sapiens OX=9606 GN=MYH9 PE=1 SV=4
Q15166	Serum paraoxonase/lactonase 3 OS=Homo sapiens OX=9606 GN=PON3 PE=1 SV=3
P12814	Alpha-actinin-1 OS=Homo sapiens OX=9606 GN=ACTN1 PE=1 SV=2
P02751	Fibronectin OS=Homo sapiens OX=9606 GN=FN1 PE=1 SV=5
P20742	Pregnancy zone protein OS=Homo sapiens OX=9606 GN=PZP PE=1 SV=4
P05155	Plasma protease C1 inhibitor OS=Homo sapiens OX=9606 GN=SERPING1 PE=1 SV=2
P11142	Heat shock cognate 71 kDa protein OS=Homo sapiens OX=9606 GN=HSPA8 PE=1 SV=1
P02765	Alpha-2-HS-glycoprotein OS=Homo sapiens OX=9606 GN=AHSG PE=1 SV=2
P43652	Afamin OS=Homo sapiens OX=9606 GN=AFM PE=1 SV=1
P11021	Endoplasmic reticulum chaperone BiP OS=Homo sapiens OX=9606 GN=HSPA5 PE=1 SV=2
P19827	Inter-alpha-trypsin inhibitor heavy chain H1 OS=Homo sapiens OX=9606 GN=ITIH1 PE=1 SV=3
P02748	Complement component C9 OS=Homo sapiens OX=9606 GN=C9 PE=1 SV=2
P29622	Kallistatin OS=Homo sapiens OX=9606 GN=SERPINA4 PE=1 SV=3
P06733	Alpha-enolase OS=Homo sapiens OX=9606 GN=ENO1 PE=1 SV=2
P02671	Fibrinogen alpha chain OS=Homo sapiens OX=9606 GN=FGA PE=1 SV=2
P00751	Complement factor B OS=Homo sapiens OX=9606 GN=CFB PE=1 SV=2
P19823	Inter-alpha-trypsin inhibitor heavy chain H2 OS=Homo sapiens OX=9606 GN=ITIH2 PE=1 SV=2

P36955	Pigment epithelium-derived factor OS=Homo sapiens OX=9606 GN=SERPINF1 PE=1 SV=4
P02649	Apolipoprotein E OS=Homo sapiens OX=9606 GN=APOE PE=1 SV=1
P07996	Thrombospondin-1 OS=Homo sapiens OX=9606 GN=THBS1 PE=1 SV=2
P02679	Fibrinogen gamma chain OS=Homo sapiens OX=9606 GN=FGG PE=1 SV=3
P02747	Complement C1q subcomponent subunit C OS=Homo sapiens OX=9606 GN=C1QC PE=1 SV=3
P18206	Vinculin OS=Homo sapiens OX=9606 GN=VCL PE=1 SV=4
P01861	Immunoglobulin heavy constant gamma 4 OS=Homo sapiens OX=9606 GN=IGHG4 PE=1 SV=1
Q13418	Integrin-linked protein kinase OS=Homo sapiens OX=9606 GN=ILK PE=1 SV=2
P01024	Complement C3 OS=Homo sapiens OX=9606 GN=C3 PE=1 SV=2
P04003	C4b-binding protein alpha chain OS=Homo sapiens OX=9606 GN=C4BPA PE=1 SV=2
P01011	Alpha-1-antichymotrypsin OS=Homo sapiens OX=9606 GN=SERPINA3 PE=1 SV=2
P10643	Complement component C7 OS=Homo sapiens OX=9606 GN=C7 PE=1 SV=2
P14625	Endoplasmin OS=Homo sapiens OX=9606 GN=HSP90B1 PE=1 SV=1
P00747	Plasminogen OS=Homo sapiens OX=9606 GN=PLG PE=1 SV=2
P31146	Coronin-1A OS=Homo sapiens OX=9606 GN=CORO1A PE=1 SV=4
P08697	Alpha-2-antiplasmin OS=Homo sapiens OX=9606 GN=SERPINF2 PE=1 SV=3
P01876	Immunoglobulin heavy constant alpha 1 OS=Homo sapiens OX=9606 GN=IGHA1 PE=1 SV=2
P13671	Complement component C6 OS=Homo sapiens OX=9606 GN=C6 PE=1 SV=3
P07737	Profilin-1 OS=Homo sapiens OX=9606 GN=PFN1 PE=1 SV=2
P10909	Clusterin OS=Homo sapiens OX=9606 GN=CLU PE=1 SV=1
P26038	Moesin OS=Homo sapiens OX=9606 GN=MSN PE=1 SV=3
P52566	Rho GDP-dissociation inhibitor 2 OS=Homo sapiens OX=9606 GN=ARHGDIB PE=1 SV=3
P06681	Complement C2 OS=Homo sapiens OX=9606 GN=C2 PE=1 SV=2
P22792	Carboxypeptidase N subunit 2 OS=Homo sapiens OX=9606 GN=CPN2 PE=1 SV=3
P50552	Vasodilator-stimulated phosphoprotein OS=Homo sapiens OX=9606 GN=VASP PE=1 SV=3
P03952	Plasma kallikrein OS=Homo sapiens OX=9606 GN=KLKB1 PE=1 SV=1
P07357	Complement component C8 alpha chain OS=Homo sapiens OX=9606 GN=C8A PE=1 SV=2
P00739	Haptoglobin-related protein OS=Homo sapiens OX=9606 GN=HPR PE=2 SV=2
P37802	Transgelin-2 OS=Homo sapiens OX=9606 GN=TAGLN2 PE=1 SV=3
P05106	Integrin beta-3 OS=Homo sapiens OX=9606 GN=ITGB3 PE=1 SV=2
P15169	Carboxypeptidase N catalytic chain OS=Homo sapiens OX=9606 GN=CPN1 PE=1 SV=1
Q04917	14-3-3 protein eta OS=Homo sapiens OX=9606 GN=YWHAH PE=1 SV=4
P27105	Erythrocyte band 7 integral membrane protein OS=Homo sapiens OX=9606 GN=STOM PE=1 SV=3
P12931	Proto-oncogene tyrosine-protein kinase Src OS=Homo sapiens OX=9606 GN=SRC PE=1 SV=3
P63104	14-3-3 protein zeta/delta OS=Homo sapiens OX=9606 GN=YWHAZ PE=1 SV=1
P06727	Apolipoprotein A-IV OS=Homo sapiens OX=9606 GN=APOA4 PE=1 SV=3
P05546	Heparin cofactor 2 OS=Homo sapiens OX=9606 GN=SERPIND1 PE=1 SV=3
P67936	Tropomyosin alpha-4 chain OS=Homo sapiens OX=9606 GN=TPM4 PE=1 SV=3
Q01518	Adenylyl cyclase-associated protein 1 OS=Homo sapiens OX=9606 GN=CAP1 PE=1 SV=5
O43866	CD5 antigen-like OS=Homo sapiens OX=9606 GN=CD5L PE=1 SV=1
P06744	Glucose-6-phosphate isomerase OS=Homo sapiens OX=9606 GN=GPI PE=1 SV=4
P21926	CD9 antigen OS=Homo sapiens OX=9606 GN=CD9 PE=1 SV=4
P04196	Histidine-rich glycoprotein OS=Homo sapiens OX=9606 GN=HRG PE=1 SV=1
Q9UBW5	Bridging integrator 2 OS=Homo sapiens OX=9606 GN=BIN2 PE=1 SV=3

A0A0B4J1V0	Immunoglobulin heavy variable 3-15 OS=Homo sapiens OX=9606 GN=IGHV3-15 PE=3 SV=1
P02750	Leucine-rich alpha-2-glycoprotein OS=Homo sapiens OX=9606 GN=LRG1 PE=1 SV=2
Q16610	Extracellular matrix protein 1 OS=Homo sapiens OX=9606 GN=ECM1 PE=1 SV=2
P23142	Fibulin-1 OS=Homo sapiens OX=9606 GN=FBLN1 PE=1 SV=4
O00299	Chloride intracellular channel protein 1 OS=Homo sapiens OX=9606 GN=CLIC1 PE=1 SV=4
P01042	Kininogen-1 OS=Homo sapiens OX=9606 GN=KNG1 PE=1 SV=2
P04275	von Willebrand factor OS=Homo sapiens OX=9606 GN=VWF PE=1 SV=4
P69905	Hemoglobin subunit alpha OS=Homo sapiens OX=9606 GN=HBA1 PE=1 SV=2
P0C0L5	Complement C4-B OS=Homo sapiens OX=9606 GN=C4B PE=1 SV=2
P04114	Apolipoprotein B-100 OS=Homo sapiens OX=9606 GN=APOB PE=1 SV=2
P0C0L4	Complement C4-A OS=Homo sapiens OX=9606 GN=C4A PE=1 SV=2
P01860	Immunoglobulin heavy constant gamma 3 OS=Homo sapiens OX=9606 GN=IGHG3 PE=1 SV=2
P08185	Corticosteroid-binding globulin OS=Homo sapiens OX=9606 GN=SERPINA6 PE=1 SV=1
P08246	Neutrophil elastase OS=Homo sapiens OX=9606 GN=ELANE PE=1 SV=1
P00488	Coagulation factor XIII A chain OS=Homo sapiens OX=9606 GN=F13A1 PE=1 SV=4
P02745	Complement C1q subcomponent subunit A OS=Homo sapiens OX=9606 GN=C1QA PE=1 SV=2
P08571	Monocyte differentiation antigen CD14 OS=Homo sapiens OX=9606 GN=CD14 PE=1 SV=2
O14791	Apolipoprotein L1 OS=Homo sapiens OX=9606 GN=APOL1 PE=1 SV=5
P60174	Triosephosphate isomerase OS=Homo sapiens OX=9606 GN=TPI1 PE=1 SV=3
P12259	Coagulation factor V OS=Homo sapiens OX=9606 GN=F5 PE=1 SV=4
P00740	Coagulation factor IX OS=Homo sapiens OX=9606 GN=F9 PE=1 SV=2
Q13201	Multimerin-1 OS=Homo sapiens OX=9606 GN=MMRN1 PE=1 SV=3
Q2TV78	Putative macrophage stimulating 1-like protein OS=Homo sapiens OX=9606 GN=MST1L PE=2 SV=2
P00918	Carbonic anhydrase 2 OS=Homo sapiens OX=9606 GN=CA2 PE=1 SV=2
O95810	Caveolae-associated protein 2 OS=Homo sapiens OX=9606 GN=CAVIN2 PE=1 SV=3
P0DOX8	Immunoglobulin lambda-1 light chain OS=Homo sapiens OX=9606 PE=1 SV=1
P48059	LIM and senescent cell antigen-like-containing domain protein 1 OS=Homo sapiens OX=9606 GN=LIMS1 PE=1 SV=4
P61224	Ras-related protein Rap-1b OS=Homo sapiens OX=9606 GN=RAP1B PE=1 SV=1
P59665	Neutrophil defensin 1 OS=Homo sapiens OX=9606 GN=DEFA1 PE=1 SV=1
P0DOY3	Immunoglobulin lambda constant 3 OS=Homo sapiens OX=9606 GN=IGLC3 PE=1 SV=1
O75636	Ficolin-3 OS=Homo sapiens OX=9606 GN=FCN3 PE=1 SV=2
P02647	Apolipoprotein A-I OS=Homo sapiens OX=9606 GN=APOA1 PE=1 SV=1
P62937	Peptidyl-prolyl cis-trans isomerase A OS=Homo sapiens OX=9606 GN=PPIA PE=1 SV=2
P68871	Hemoglobin subunit beta OS=Homo sapiens OX=9606 GN=HBB PE=1 SV=2
P35542	Serum amyloid A-4 protein OS=Homo sapiens OX=9606 GN=SAA4 PE=1 SV=2
P61158	Actin-related protein 3 OS=Homo sapiens OX=9606 GN=ACTR3 PE=1 SV=3
P20851	C4b-binding protein beta chain OS=Homo sapiens OX=9606 GN=C4BPB PE=1 SV=1
P33908	Mannosyl-oligosaccharide 1,2-alpha-mannosidase IA OS=Homo sapiens OX=9606 GN=MAN1A1 PE=1 SV=3
P27797	Calreticulin OS=Homo sapiens OX=9606 GN=CALR PE=1 SV=1
P23528	Cofilin-1 OS=Homo sapiens OX=9606 GN=CFL1 PE=1 SV=3
P62491	Ras-related protein Rab-11A OS=Homo sapiens OX=9606 GN=RAB11A PE=1 SV=3

P08519	Apolipoprotein(a) OS=Homo sapiens OX=9606 GN=LPA PE=1 SV=1
P61160	Actin-related protein 2 OS=Homo sapiens OX=9606 GN=ACTR2 PE=1 SV=1
P78417	Glutathione S-transferase omega-1 OS=Homo sapiens OX=9606 GN=GSTO1 PE=1 SV=2
P49908	Selenoprotein P OS=Homo sapiens OX=9606 GN=SELENOP PE=1 SV=3
P31946	14-3-3 protein beta/alpha OS=Homo sapiens OX=9606 GN=YWHAB PE=1 SV=3
P11413	Glucose-6-phosphate 1-dehydrogenase OS=Homo sapiens OX=9606 GN=G6PD PE=1 SV=4
P02766	Transthyretin OS=Homo sapiens OX=9606 GN=TTR PE=1 SV=1
O95445	Apolipoprotein M OS=Homo sapiens OX=9606 GN=APOM PE=1 SV=2
P04278	Sex hormone-binding globulin OS=Homo sapiens OX=9606 GN=SHBG PE=1 SV=2
P04259	Keratin, type II cytoskeletal 6B OS=Homo sapiens OX=9606 GN=KRT6B PE=1 SV=5
P08779	Keratin, type I cytoskeletal 16 OS=Homo sapiens OX=9606 GN=KRT16 PE=1 SV=4
A0A075B6I9	Immunoglobulin lambda variable 7-46 OS=Homo sapiens OX=9606 GN=IGLV7-46 PE=3 SV=4
P63261	Actin, cytoplasmic 2 OS=Homo sapiens OX=9606 GN=ACTG1 PE=1 SV=1
P61026	Ras-related protein Rab-10 OS=Homo sapiens OX=9606 GN=RAB10 PE=1 SV=1
P02533	Keratin, type I cytoskeletal 14 OS=Homo sapiens OX=9606 GN=KRT14 PE=1 SV=4
P0DOX6	Immunoglobulin mu heavy chain OS=Homo sapiens OX=9606 PE=1 SV=2
Q04695	Keratin, type I cytoskeletal 17 OS=Homo sapiens OX=9606 GN=KRT17 PE=1 SV=2
Q5D862	Filaggrin-2 OS=Homo sapiens OX=9606 GN=FLG2 PE=1 SV=1
P01019	Angiotensinogen OS=Homo sapiens OX=9606 GN=AGT PE=1 SV=1
P0DOX2	Immunoglobulin alpha-2 heavy chain OS=Homo sapiens OX=9606 PE=1 SV=2
Q9UK55	Protein Z-dependent protease inhibitor OS=Homo sapiens OX=9606 GN=SERPINA10 PE=1 SV=1
Q86YZ3	Hornerin OS=Homo sapiens OX=9606 GN=HRNR PE=1 SV=2
Q5T749	Keratinocyte proline-rich protein OS=Homo sapiens OX=9606 GN=KPRP PE=1 SV=1
P29508	Serpin B3 OS=Homo sapiens OX=9606 GN=SERPINB3 PE=1 SV=2
P05109	Protein S100-A8 OS=Homo sapiens OX=9606 GN=S100A8 PE=1 SV=1
P02538	Keratin, type II cytoskeletal 6A OS=Homo sapiens OX=9606 GN=KRT6A PE=1 SV=3
Q14766	Latent-transforming growth factor beta-binding protein 1 OS=Homo sapiens OX=9606 GN=LTBP1 PE=1 SV=4
P02787	Serotransferrin OS=Homo sapiens OX=9606 GN=TF PE=1 SV=3
P01619	Immunoglobulin kappa variable 3-20 OS=Homo sapiens OX=9606 GN=IGKV3-20 PE=1 SV=2
P14923	Junction plakoglobin OS=Homo sapiens OX=9606 GN=JUP PE=1 SV=3
P15924	Desmoplakin OS=Homo sapiens OX=9606 GN=DSP PE=1 SV=3
Q3SY84	Keratin, type II cytoskeletal 71 OS=Homo sapiens OX=9606 GN=KRT71 PE=1 SV=3
Q14520	Hyaluronan-binding protein 2 OS=Homo sapiens OX=9606 GN=HABP2 PE=1 SV=1
Q07960	Rho GTPase-activating protein 1 OS=Homo sapiens OX=9606 GN=ARHGAP1 PE=1 SV=1
Q15404	Ras suppressor protein 1 OS=Homo sapiens OX=9606 GN=RSU1 PE=1 SV=3
P61981	14-3-3 protein gamma OS=Homo sapiens OX=9606 GN=YWHAG PE=1 SV=2
Q6KB66	Keratin, type II cytoskeletal 80 OS=Homo sapiens OX=9606 GN=KRT80 PE=1 SV=2
Q01813	ATP-dependent 6-phosphofructokinase, platelet type OS=Homo sapiens OX=9606 GN=PFKP PE=1 SV=2
O76011	Keratin, type I cuticular Ha4 OS=Homo sapiens OX=9606 GN=KRT34 PE=1 SV=2
P08311	Cathepsin G OS=Homo sapiens OX=9606 GN=CTSG PE=1 SV=2
P0DMV8	Heat shock 70 kDa protein 1A OS=Homo sapiens OX=9606 GN=HSPA1A PE=1 SV=1
P37837	Transaldolase OS=Homo sapiens OX=9606 GN=TALDO1 PE=1 SV=2

Q14019	Coactosin-like protein OS=Homo sapiens OX=9606 GN=COTL1 PE=1 SV=3
Q14525	Keratin, type I cuticular Ha3-II OS=Homo sapiens OX=9606 GN=KRT33B PE=1 SV=3
P78371	T-complex protein 1 subunit beta OS=Homo sapiens OX=9606 GN=CCT2 PE=1 SV=4
A0A0B4J1X5	Immunoglobulin heavy variable 3-74 OS=Homo sapiens OX=9606 GN=IGHV3-74 PE=3 SV=1
Q15084	Protein disulfide-isomerase A6 OS=Homo sapiens OX=9606 GN=PDIA6 PE=1 SV=1
P31150	Rab GDP dissociation inhibitor alpha OS=Homo sapiens OX=9606 GN=GDI1 PE=1 SV=2
P68371	Tubulin beta-4B chain OS=Homo sapiens OX=9606 GN=TUBB4B PE=1 SV=1
P04180	Phosphatidylcholine-sterol acyltransferase OS=Homo sapiens OX=9606 GN=LCAT PE=1 SV=1
P30740	Leukocyte elastase inhibitor OS=Homo sapiens OX=9606 GN=SERPINB1 PE=1 SV=1
P07359	Platelet glycoprotein Ib alpha chain OS=Homo sapiens OX=9606 GN=GP1BA PE=1 SV=2
Q9BR76	Coronin-1B OS=Homo sapiens OX=9606 GN=CORO1B PE=1 SV=1
P04075	Fructose-bisphosphate aldolase A OS=Homo sapiens OX=9606 GN=ALDOA PE=1 SV=2
P80748	Immunoglobulin lambda variable 3-21 OS=Homo sapiens OX=9606 GN=IGLV3-21 PE=1 SV=2
P55209	Nucleosome assembly protein 1-like 1 OS=Homo sapiens OX=9606 GN=NAP1L1 PE=1 SV=1
P07900	Heat shock protein HSP 90-alpha OS=Homo sapiens OX=9606 GN=HSP90AA1 PE=1 SV=5
P08729	Keratin, type II cytoskeletal 7 OS=Homo sapiens OX=9606 GN=KRT7 PE=1 SV=5
Q5JSH3	WD repeat-containing protein 44 OS=Homo sapiens OX=9606 GN=WDR44 PE=1 SV=1
P30041	Peroxiredoxin-6 OS=Homo sapiens OX=9606 GN=PRDX6 PE=1 SV=3
Q9NZN3	EH domain-containing protein 3 OS=Homo sapiens OX=9606 GN=EHD3 PE=1 SV=2
P06702	Protein S100-A9 OS=Homo sapiens OX=9606 GN=S100A9 PE=1 SV=1
P01780	Immunoglobulin heavy variable 3-7 OS=Homo sapiens OX=9606 GN=IGHV3-7 PE=1 SV=2
P01768	Immunoglobulin heavy variable 3-30 OS=Homo sapiens OX=9606 GN=IGHV3-30 PE=1 SV=2
O15143	Actin-related protein 2/3 complex subunit 1B OS=Homo sapiens OX=9606 GN=ARPC1B PE=1 SV=3
P48741	Putative heat shock 70 kDa protein 7 OS=Homo sapiens OX=9606 GN=HSPA7 PE=5 SV=2
P60660	Myosin light polypeptide 6 OS=Homo sapiens OX=9606 GN=MYL6 PE=1 SV=2
Q15942	Zyxin OS=Homo sapiens OX=9606 GN=ZYX PE=1 SV=1
P81605	Dermcidin OS=Homo sapiens OX=9606 GN=DCD PE=1 SV=2
P01880	Immunoglobulin heavy constant delta OS=Homo sapiens OX=9606 GN=IGHD PE=1 SV=3
P01591	Immunoglobulin J chain OS=Homo sapiens OX=9606 GN=JCHAIN PE=1 SV=4
P13224	Platelet glycoprotein Ib beta chain OS=Homo sapiens OX=9606 GN=GP1BB PE=1 SV=1
P48509	CD151 antigen OS=Homo sapiens OX=9606 GN=CD151 PE=1 SV=3
P0DOX7	Immunoglobulin kappa light chain OS=Homo sapiens OX=9606 PE=1 SV=1
P23284	Peptidyl-prolyl cis-trans isomerase B OS=Homo sapiens OX=9606 GN=PPIB PE=1 SV=2
P15153	Ras-related C3 botulinum toxin substrate 2 OS=Homo sapiens OX=9606 GN=RAC2 PE=1 SV=1
P05452	Tetranectin OS=Homo sapiens OX=9606 GN=CLEC3B PE=1 SV=3
P49747	Cartilage oligomeric matrix protein OS=Homo sapiens OX=9606 GN=COMP PE=1 SV=2
P61204	ADP-ribosylation factor 3 OS=Homo sapiens OX=9606 GN=ARF3 PE=1 SV=2
P60953	Cell division control protein 42 homolog OS=Homo sapiens OX=9606 GN=CDC42 PE=1 SV=2
P02652	Apolipoprotein A-II OS=Homo sapiens OX=9606 GN=APOA2 PE=1 SV=1
P01717	Immunoglobulin lambda variable 3-25 OS=Homo sapiens OX=9606 GN=IGLV3-25 PE=1 SV=2
Q92954	Proteoglycan 4 OS=Homo sapiens OX=9606 GN=PRG4 PE=1 SV=3
O43790	Keratin, type II cuticular Hb6 OS=Homo sapiens OX=9606 GN=KRT86 PE=1 SV=1
P18428	Lipopolysaccharide-binding protein OS=Homo sapiens OX=9606 GN=LBP PE=1 SV=3

P00915	Carbonic anhydrase 1 OS=Homo sapiens OX=9606 GN=CA1 PE=1 SV=2
Q16181	Septin-7 OS=Homo sapiens OX=9606 GN=SEPTIN7 PE=1 SV=2
P08238	Heat shock protein HSP 90-beta OS=Homo sapiens OX=9606 GN=HSP90AB1 PE=1 SV=4
Q8IUC1	Keratin-associated protein 11-1 OS=Homo sapiens OX=9606 GN=KRTAP11-1 PE=2 SV=1
Q7Z3Y7	Keratin, type I cytoskeletal 28 OS=Homo sapiens OX=9606 GN=KRT28 PE=1 SV=2
P27348	14-3-3 protein theta OS=Homo sapiens OX=9606 GN=YWHAQ PE=1 SV=1
O76013	Keratin, type I cuticular Ha6 OS=Homo sapiens OX=9606 GN=KRT36 PE=2 SV=1
Q15323	Keratin, type I cuticular Ha1 OS=Homo sapiens OX=9606 GN=KRT31 PE=1 SV=3
Q15485	Ficolin-2 OS=Homo sapiens OX=9606 GN=FCN2 PE=1 SV=2
A6NCN2	Putative keratin-87 protein OS=Homo sapiens OX=9606 GN=KRT87P PE=5 SV=4
O15144	Actin-related protein 2/3 complex subunit 2 OS=Homo sapiens OX=9606 GN=ARPC2 PE=1 SV=1
P78386	Keratin, type II cuticular Hb5 OS=Homo sapiens OX=9606 GN=KRT85 PE=1 SV=1
Q9NSB4	Keratin, type II cuticular Hb2 OS=Homo sapiens OX=9606 GN=KRT82 PE=3 SV=3
P20930	Filaggrin OS=Homo sapiens OX=9606 GN=FLG PE=1 SV=3
Q9NZP8	Complement C1r subcomponent-like protein OS=Homo sapiens OX=9606 GN=C1RL PE=1 SV=2
A0A075B6K4	Immunoglobulin lambda variable 3-10 OS=Homo sapiens OX=9606 GN=IGLV3-10 PE=3 SV=2
P19652	Alpha-1-acid glycoprotein 2 OS=Homo sapiens OX=9606 GN=ORM2 PE=1 SV=2
P01709	Immunoglobulin lambda variable 2-8 OS=Homo sapiens OX=9606 GN=IGLV2-8 PE=1 SV=2
Q92764	Keratin, type I cuticular Ha5 OS=Homo sapiens OX=9606 GN=KRT35 PE=2 SV=5

Supplementary Table 3. Protein profile obtained for OSP.

Accesion number	Description
P60709	Actin, cytoplasmic 1 OS=Homo sapiens OX=9606 GN=ACTB PE=1 SV=1
P07437	Tubulin beta chain OS=Homo sapiens OX=9606 GN=TUBB PE=1 SV=2
P68133	Actin, alpha skeletal muscle OS=Homo sapiens OX=9606 GN=ACTA1 PE=1 SV=1
P01834	Immunoglobulin kappa constant OS=Homo sapiens OX=9606 GN=IGKC PE=1 SV=2
P04264	Keratin, type II cytoskeletal 1 OS=Homo sapiens OX=9606 GN=KRT1 PE=1 SV=6
P35527	Keratin, type I cytoskeletal 9 OS=Homo sapiens OX=9606 GN=KRT9 PE=1 SV=3
P02768	Serum albumin OS=Homo sapiens OX=9606 GN=ALB PE=1 SV=2
P68366	Tubulin alpha-4A chain OS=Homo sapiens OX=9606 GN=TUBA4A PE=1 SV=1
Q9HBI1	Beta-parvin OS=Homo sapiens OX=9606 GN=PARVB PE=1 SV=1
P01857	Immunoglobulin heavy constant gamma 1 OS=Homo sapiens OX=9606 GN=IGHG1 PE=1 SV=1
P35908	Keratin, type II cytoskeletal 2 epidermal OS=Homo sapiens OX=9606 GN=KRT2 PE=1 SV=2
P14618	Pyruvate kinase PKM OS=Homo sapiens OX=9606 GN=PKM PE=1 SV=4
P01008	Antithrombin-III OS=Homo sapiens OX=9606 GN=SERPINC1 PE=1 SV=1
P00450	Ceruloplasmin OS=Homo sapiens OX=9606 GN=CP PE=1 SV=1
P04406	Glyceraldehyde-3-phosphate dehydrogenase OS=Homo sapiens OX=9606 GN=GAPDH PE=1 SV=3
P13645	Keratin, type I cytoskeletal 10 OS=Homo sapiens OX=9606 GN=KRT10 PE=1 SV=6
P27918	Properdin OS=Homo sapiens OX=9606 GN=CFP PE=1 SV=2
P04217	Alpha-1B-glycoprotein OS=Homo sapiens OX=9606 GN=A1BG PE=1 SV=4
P27169	Serum paraoxonase/arylesterase 1 OS=Homo sapiens OX=9606 GN=PON1 PE=1 SV=3
P13647	Keratin, type II cytoskeletal 5 OS=Homo sapiens OX=9606 GN=KRT5 PE=1 SV=3

Q9H4B7	Tubulin beta-1 chain OS=Homo sapiens OX=9606 GN=TUBB1 PE=1 SV=1
Q96PD5	N-acetylmuramoyl-L-alanine amidase OS=Homo sapiens OX=9606 GN=PGLYRP2 PE=1 SV=1
P00558	Phosphoglycerate kinase 1 OS=Homo sapiens OX=9606 GN=PGK1 PE=1 SV=3
P68363	Tubulin alpha-1B chain OS=Homo sapiens OX=9606 GN=TUBA1B PE=1 SV=1
Q96IY4	Carboxypeptidase B2 OS=Homo sapiens OX=9606 GN=CPB2 PE=1 SV=2
P00734	Prothrombin OS=Homo sapiens OX=9606 GN=F2 PE=1 SV=2
Q9Y490	Talin-1 OS=Homo sapiens OX=9606 GN=TLN1 PE=1 SV=3
P08603	Complement factor H OS=Homo sapiens OX=9606 GN=CFH PE=1 SV=4
P07360	Complement component C8 gamma chain OS=Homo sapiens OX=9606 GN=C8G PE=1 SV=3
Q562R1	Beta-actin-like protein 2 OS=Homo sapiens OX=9606 GN=ACTBL2 PE=1 SV=2
P52907	F-actin-capping protein subunit alpha-1 OS=Homo sapiens OX=9606 GN=CAPZA1 PE=1 SV=3
P07225	Vitamin K-dependent protein S OS=Homo sapiens OX=9606 GN=PROS1 PE=1 SV=1
P02675	Fibrinogen beta chain OS=Homo sapiens OX=9606 GN=FGB PE=1 SV=2
P30101	Protein disulfide-isomerase A3 OS=Homo sapiens OX=9606 GN=PDIA3 PE=1 SV=4
P06396	Gelsolin OS=Homo sapiens OX=9606 GN=GSN PE=1 SV=1
P01031	Complement C5 OS=Homo sapiens OX=9606 GN=C5 PE=1 SV=4
P02790	Hemopexin OS=Homo sapiens OX=9606 GN=HPX PE=1 SV=2
P08514	Integrin alpha-IIb OS=Homo sapiens OX=9606 GN=ITGA2B PE=1 SV=3
Q14624	Inter-alpha-trypsin inhibitor heavy chain H4 OS=Homo sapiens OX=9606 GN=ITIH4 PE=1 SV=4
P02760	Protein AMBP OS=Homo sapiens OX=9606 GN=AMBP PE=1 SV=1
P48740	Mannan-binding lectin serine protease 1 OS=Homo sapiens OX=9606 GN=MASP1 PE=1 SV=3
P01009	Alpha-1-antitrypsin OS=Homo sapiens OX=9606 GN=SERPINA1 PE=1 SV=3
P01859	Immunoglobulin heavy constant gamma 2 OS=Homo sapiens OX=9606 GN=IGHG2 PE=1 SV=2
P00738	Haptoglobin OS=Homo sapiens OX=9606 GN=HP PE=1 SV=1
P35858	Insulin-like growth factor-binding protein complex acid labile subunit OS=Homo sapiens OX=9606 GN=IGFALS PE=1 SV=1
P40197	Platelet glycoprotein V OS=Homo sapiens OX=9606 GN=GP5 PE=1 SV=1
P08567	Pleckstrin OS=Homo sapiens OX=9606 GN=PLEK PE=1 SV=3
O75083	WD repeat-containing protein 1 OS=Homo sapiens OX=9606 GN=WDR1 PE=1 SV=4
P01023	Alpha-2-macroglobulin OS=Homo sapiens OX=9606 GN=A2M PE=1 SV=3
Q9ULV4	Coronin-1C OS=Homo sapiens OX=9606 GN=CORO1C PE=1 SV=1
Q06033	Inter-alpha-trypsin inhibitor heavy chain H3 OS=Homo sapiens OX=9606 GN=ITIH3 PE=1 SV=2
P04004	Vitronectin OS=Homo sapiens OX=9606 GN=VTN PE=1 SV=1
P02746	Complement C1q subcomponent subunit B OS=Homo sapiens OX=9606 GN=C1QB PE=1 SV=3
P51884	Lumican OS=Homo sapiens OX=9606 GN=LUM PE=1 SV=2
P07358	Complement component C8 beta chain OS=Homo sapiens OX=9606 GN=C8B PE=1 SV=3
P02749	Beta-2-glycoprotein 1 OS=Homo sapiens OX=9606 GN=APOH PE=1 SV=3
P02774	Vitamin D-binding protein OS=Homo sapiens OX=9606 GN=GC PE=1 SV=2
Q86UX7	Fermitin family homolog 3 OS=Homo sapiens OX=9606 GN=FERMT3 PE=1 SV=1
P00736	Complement C1r subcomponent OS=Homo sapiens OX=9606 GN=C1R PE=1 SV=2
P21333	Filamin-A OS=Homo sapiens OX=9606 GN=FLNA PE=1 SV=4
P01871	Immunoglobulin heavy constant mu OS=Homo sapiens OX=9606 GN=IGHM PE=1 SV=4
P00742	Coagulation factor X OS=Homo sapiens OX=9606 GN=F10 PE=1 SV=2
P09871	Complement C1s subcomponent OS=Homo sapiens OX=9606 GN=C1S PE=1 SV=1

P35579	Myosin-9 OS=Homo sapiens OX=9606 GN=MYH9 PE=1 SV=4
Q15166	Serum paraoxonase/lactonase 3 OS=Homo sapiens OX=9606 GN=PON3 PE=1 SV=3
P12814	Alpha-actinin-1 OS=Homo sapiens OX=9606 GN=ACTN1 PE=1 SV=2
P02751	Fibronectin OS=Homo sapiens OX=9606 GN=FN1 PE=1 SV=5
P20742	Pregnancy zone protein OS=Homo sapiens OX=9606 GN=PZP PE=1 SV=4
P05155	Plasma protease C1 inhibitor OS=Homo sapiens OX=9606 GN=SERPING1 PE=1 SV=2
P11142	Heat shock cognate 71 kDa protein OS=Homo sapiens OX=9606 GN=HSPA8 PE=1 SV=1
P02765	Alpha-2-HS-glycoprotein OS=Homo sapiens OX=9606 GN=AHSG PE=1 SV=2
P43652	Afamin OS=Homo sapiens OX=9606 GN=AFM PE=1 SV=1
P11021	Endoplasmic reticulum chaperone BiP OS=Homo sapiens OX=9606 GN=HSPA5 PE=1 SV=2
P19827	Inter-alpha-trypsin inhibitor heavy chain H1 OS=Homo sapiens OX=9606 GN=ITIH1 PE=1 SV=3
P02748	Complement component C9 OS=Homo sapiens OX=9606 GN=C9 PE=1 SV=2
P29622	Kallistatin OS=Homo sapiens OX=9606 GN=SERPINA4 PE=1 SV=3
P06733	Alpha-enolase OS=Homo sapiens OX=9606 GN=ENO1 PE=1 SV=2
P02671	Fibrinogen alpha chain OS=Homo sapiens OX=9606 GN=FGA PE=1 SV=2
P00751	Complement factor B OS=Homo sapiens OX=9606 GN=CFB PE=1 SV=2
P19823	Inter-alpha-trypsin inhibitor heavy chain H2 OS=Homo sapiens OX=9606 GN=ITIH2 PE=1 SV=2
P36955	Pigment epithelium-derived factor OS=Homo sapiens OX=9606 GN=SERPINF1 PE=1 SV=4
P02649	Apolipoprotein E OS=Homo sapiens OX=9606 GN=APOE PE=1 SV=1
P07996	Thrombospondin-1 OS=Homo sapiens OX=9606 GN=THBS1 PE=1 SV=2
P02679	Fibrinogen gamma chain OS=Homo sapiens OX=9606 GN=FGG PE=1 SV=3
P02747	Complement C1q subcomponent subunit C OS=Homo sapiens OX=9606 GN=C1QC PE=1 SV=3
P18206	Vinculin OS=Homo sapiens OX=9606 GN=VCL PE=1 SV=4
P01861	Immunoglobulin heavy constant gamma 4 OS=Homo sapiens OX=9606 GN=IGHG4 PE=1 SV=1
Q13418	Integrin-linked protein kinase OS=Homo sapiens OX=9606 GN=ILK PE=1 SV=2
P01024	Complement C3 OS=Homo sapiens OX=9606 GN=C3 PE=1 SV=2
P04003	C4b-binding protein alpha chain OS=Homo sapiens OX=9606 GN=C4BPA PE=1 SV=2
P01011	Alpha-1-antichymotrypsin OS=Homo sapiens OX=9606 GN=SERPINA3 PE=1 SV=2
P10643	Complement component C7 OS=Homo sapiens OX=9606 GN=C7 PE=1 SV=2
P14625	Endoplasmin OS=Homo sapiens OX=9606 GN=HSP90B1 PE=1 SV=1
P00747	Plasminogen OS=Homo sapiens OX=9606 GN=PLG PE=1 SV=2
P31146	Coronin-1A OS=Homo sapiens OX=9606 GN=CORO1A PE=1 SV=4
P08697	Alpha-2-antiplasmin OS=Homo sapiens OX=9606 GN=SERPINF2 PE=1 SV=3
P01876	Immunoglobulin heavy constant alpha 1 OS=Homo sapiens OX=9606 GN=IGHA1 PE=1 SV=2
P13671	Complement component C6 OS=Homo sapiens OX=9606 GN=C6 PE=1 SV=3
P07737	Profilin-1 OS=Homo sapiens OX=9606 GN=PFN1 PE=1 SV=2
P10909	Clusterin OS=Homo sapiens OX=9606 GN=CLU PE=1 SV=1
P26038	Moesin OS=Homo sapiens OX=9606 GN=MSN PE=1 SV=3
P52566	Rho GDP-dissociation inhibitor 2 OS=Homo sapiens OX=9606 GN=ARHGDIB PE=1 SV=3
P06681	Complement C2 OS=Homo sapiens OX=9606 GN=C2 PE=1 SV=2
P22792	Carboxypeptidase N subunit 2 OS=Homo sapiens OX=9606 GN=CPN2 PE=1 SV=3
P50552	Vasodilator-stimulated phosphoprotein OS=Homo sapiens OX=9606 GN=VASP PE=1 SV=3
P03952	Plasma kallikrein OS=Homo sapiens OX=9606 GN=KLKB1 PE=1 SV=1
P07357	Complement component C8 alpha chain OS=Homo sapiens OX=9606 GN=C8A PE=1 SV=2

P00739	Haptoglobin-related protein OS=Homo sapiens OX=9606 GN=HPR PE=2 SV=2
P37802	Transgelin-2 OS=Homo sapiens OX=9606 GN=TAGLN2 PE=1 SV=3
P05106	Integrin beta-3 OS=Homo sapiens OX=9606 GN=ITGB3 PE=1 SV=2
P15169	Carboxypeptidase N catalytic chain OS=Homo sapiens OX=9606 GN=CPN1 PE=1 SV=1
Q04917	14-3-3 protein eta OS=Homo sapiens OX=9606 GN=YWHAH PE=1 SV=4
P27105	Erythrocyte band 7 integral membrane protein OS=Homo sapiens OX=9606 GN=STOM PE=1 SV=3
P12931	Proto-oncogene tyrosine-protein kinase Src OS=Homo sapiens OX=9606 GN=SRC PE=1 SV=3
P63104	14-3-3 protein zeta/delta OS=Homo sapiens OX=9606 GN=YWHAZ PE=1 SV=1
P06727	Apolipoprotein A-IV OS=Homo sapiens OX=9606 GN=APOA4 PE=1 SV=3
P05546	Heparin cofactor 2 OS=Homo sapiens OX=9606 GN=SERPIND1 PE=1 SV=3
P67936	Tropomyosin alpha-4 chain OS=Homo sapiens OX=9606 GN=TPM4 PE=1 SV=3
Q01518	Adenylyl cyclase-associated protein 1 OS=Homo sapiens OX=9606 GN=CAP1 PE=1 SV=5
O43866	CD5 antigen-like OS=Homo sapiens OX=9606 GN=CD5L PE=1 SV=1
P06744	Glucose-6-phosphate isomerase OS=Homo sapiens OX=9606 GN=GPI PE=1 SV=4
P21926	CD9 antigen OS=Homo sapiens OX=9606 GN=CD9 PE=1 SV=4
P04196	Histidine-rich glycoprotein OS=Homo sapiens OX=9606 GN=HRG PE=1 SV=1
Q9UBW5	Bridging integrator 2 OS=Homo sapiens OX=9606 GN=BIN2 PE=1 SV=3
A0A0B4J1V0	Immunoglobulin heavy variable 3-15 OS=Homo sapiens OX=9606 GN=IGHV3-15 PE=3 SV=1
P02750	Leucine-rich alpha-2-glycoprotein OS=Homo sapiens OX=9606 GN=LRG1 PE=1 SV=2
Q16610	Extracellular matrix protein 1 OS=Homo sapiens OX=9606 GN=ECM1 PE=1 SV=2
P23142	Fibulin-1 OS=Homo sapiens OX=9606 GN=FBLN1 PE=1 SV=4
O00299	Chloride intracellular channel protein 1 OS=Homo sapiens OX=9606 GN=CLIC1 PE=1 SV=4
P01042	Kininogen-1 OS=Homo sapiens OX=9606 GN=KNG1 PE=1 SV=2
P04275	von Willebrand factor OS=Homo sapiens OX=9606 GN=VWF PE=1 SV=4
P69905	Hemoglobin subunit alpha OS=Homo sapiens OX=9606 GN=HBA1 PE=1 SV=2
P0C0L5	Complement C4-B OS=Homo sapiens OX=9606 GN=C4B PE=1 SV=2
P04114	Apolipoprotein B-100 OS=Homo sapiens OX=9606 GN=APOB PE=1 SV=2
P0C0L4	Complement C4-A OS=Homo sapiens OX=9606 GN=C4A PE=1 SV=2
P01860	Immunoglobulin heavy constant gamma 3 OS=Homo sapiens OX=9606 GN=IGHG3 PE=1 SV=2
P08185	Corticosteroid-binding globulin OS=Homo sapiens OX=9606 GN=SERPINA6 PE=1 SV=1
P08246	Neutrophil elastase OS=Homo sapiens OX=9606 GN=ELANE PE=1 SV=1
P00488	Coagulation factor XIII A chain OS=Homo sapiens OX=9606 GN=F13A1 PE=1 SV=4
P02745	Complement C1q subcomponent subunit A OS=Homo sapiens OX=9606 GN=C1QA PE=1 SV=2
P08571	Monocyte differentiation antigen CD14 OS=Homo sapiens OX=9606 GN=CD14 PE=1 SV=2
O14791	Apolipoprotein L1 OS=Homo sapiens OX=9606 GN=APOL1 PE=1 SV=5
P60174	Triosephosphate isomerase OS=Homo sapiens OX=9606 GN=TPI1 PE=1 SV=3
P12259	Coagulation factor V OS=Homo sapiens OX=9606 GN=F5 PE=1 SV=4
P00740	Coagulation factor IX OS=Homo sapiens OX=9606 GN=F9 PE=1 SV=2
Q13201	Multimerin-1 OS=Homo sapiens OX=9606 GN=MMRN1 PE=1 SV=3
Q2TV78	Putative macrophage stimulating 1-like protein OS=Homo sapiens OX=9606 GN=MST1L PE=2 SV=2
P00918	Carbonic anhydrase 2 OS=Homo sapiens OX=9606 GN=CA2 PE=1 SV=2
O95810	Caveolae-associated protein 2 OS=Homo sapiens OX=9606 GN=CAVIN2 PE=1 SV=3
P0DOX8	Immunoglobulin lambda-1 light chain OS=Homo sapiens OX=9606 PE=1 SV=1

P48059	LIM and senescent cell antigen-like-containing domain protein 1 OS=Homo sapiens OX=9606 GN=LIMS1 PE=1 SV=4
P61224	Ras-related protein Rap-1b OS=Homo sapiens OX=9606 GN=RAP1B PE=1 SV=1
P59665	Neutrophil defensin 1 OS=Homo sapiens OX=9606 GN=DEFA1 PE=1 SV=1
P0DOY3	Immunoglobulin lambda constant 3 OS=Homo sapiens OX=9606 GN=IGLC3 PE=1 SV=1
O75636	Ficolin-3 OS=Homo sapiens OX=9606 GN=FCN3 PE=1 SV=2
P02647	Apolipoprotein A-I OS=Homo sapiens OX=9606 GN=APOA1 PE=1 SV=1
P62937	Peptidyl-prolyl cis-trans isomerase A OS=Homo sapiens OX=9606 GN=PPIA PE=1 SV=2
P68871	Hemoglobin subunit beta OS=Homo sapiens OX=9606 GN=HBB PE=1 SV=2
P35542	Serum amyloid A-4 protein OS=Homo sapiens OX=9606 GN=SAA4 PE=1 SV=2
P61158	Actin-related protein 3 OS=Homo sapiens OX=9606 GN=ACTR3 PE=1 SV=3
P20851	C4b-binding protein beta chain OS=Homo sapiens OX=9606 GN=C4BPB PE=1 SV=1
P33908	Mannosyl-oligosaccharide 1,2-alpha-mannosidase IA OS=Homo sapiens OX=9606 GN=MAN1A1 PE=1 SV=3
P27797	Calreticulin OS=Homo sapiens OX=9606 GN=CALR PE=1 SV=1
P23528	Cofilin-1 OS=Homo sapiens OX=9606 GN=CFL1 PE=1 SV=3
P62491	Ras-related protein Rab-11A OS=Homo sapiens OX=9606 GN=RAB11A PE=1 SV=3
P08519	Apolipoprotein(a) OS=Homo sapiens OX=9606 GN=LPA PE=1 SV=1
P61160	Actin-related protein 2 OS=Homo sapiens OX=9606 GN=ACTR2 PE=1 SV=1
P78417	Glutathione S-transferase omega-1 OS=Homo sapiens OX=9606 GN=GSTO1 PE=1 SV=2
P49908	Selenoprotein P OS=Homo sapiens OX=9606 GN=SELENOP PE=1 SV=3
P31946	14-3-3 protein beta/alpha OS=Homo sapiens OX=9606 GN=YWHAB PE=1 SV=3
P11413	Glucose-6-phosphate 1-dehydrogenase OS=Homo sapiens OX=9606 GN=G6PD PE=1 SV=4
P02766	Transthyretin OS=Homo sapiens OX=9606 GN=TTR PE=1 SV=1
O95445	Apolipoprotein M OS=Homo sapiens OX=9606 GN=APOM PE=1 SV=2
P04278	Sex hormone-binding globulin OS=Homo sapiens OX=9606 GN=SHBG PE=1 SV=2
P04259	Keratin, type II cytoskeletal 6B OS=Homo sapiens OX=9606 GN=KRT6B PE=1 SV=5
P08779	Keratin, type I cytoskeletal 16 OS=Homo sapiens OX=9606 GN=KRT16 PE=1 SV=4
A0A075B6I9	Immunoglobulin lambda variable 7-46 OS=Homo sapiens OX=9606 GN=IGLV7-46 PE=3 SV=4
P63261	Actin, cytoplasmic 2 OS=Homo sapiens OX=9606 GN=ACTG1 PE=1 SV=1
P61026	Ras-related protein Rab-10 OS=Homo sapiens OX=9606 GN=RAB10 PE=1 SV=1
P02533	Keratin, type I cytoskeletal 14 OS=Homo sapiens OX=9606 GN=KRT14 PE=1 SV=4
P0DOX6	Immunoglobulin mu heavy chain OS=Homo sapiens OX=9606 PE=1 SV=2
Q04695	Keratin, type I cytoskeletal 17 OS=Homo sapiens OX=9606 GN=KRT17 PE=1 SV=2
Q5D862	Filaggrin-2 OS=Homo sapiens OX=9606 GN=FLG2 PE=1 SV=1
P01019	Angiotensinogen OS=Homo sapiens OX=9606 GN=AGT PE=1 SV=1
P0DOX2	Immunoglobulin alpha-2 heavy chain OS=Homo sapiens OX=9606 PE=1 SV=2
Q9UK55	Protein Z-dependent protease inhibitor OS=Homo sapiens OX=9606 GN=SERPINA10 PE=1 SV=1
Q86YZ3	Hornerin OS=Homo sapiens OX=9606 GN=HRNR PE=1 SV=2
Q5T749	Keratinocyte proline-rich protein OS=Homo sapiens OX=9606 GN=KPRP PE=1 SV=1
P29508	Serpin B3 OS=Homo sapiens OX=9606 GN=SERPINB3 PE=1 SV=2
P05109	Protein S100-A8 OS=Homo sapiens OX=9606 GN=S100A8 PE=1 SV=1
P02538	Keratin, type II cytoskeletal 6A OS=Homo sapiens OX=9606 GN=KRT6A PE=1 SV=3
Q14766	Latent-transforming growth factor beta-binding protein 1 OS=Homo sapiens OX=9606 GN=LTBP1

	PE=1 SV=4
P02787	Serotransferrin OS=Homo sapiens OX=9606 GN=TF PE=1 SV=3
P01619	Immunoglobulin kappa variable 3-20 OS=Homo sapiens OX=9606 GN=IGKV3-20 PE=1 SV=2
P14923	Junction plakoglobin OS=Homo sapiens OX=9606 GN=JUP PE=1 SV=3
P15924	Desmoplakin OS=Homo sapiens OX=9606 GN=DSP PE=1 SV=3
Q3SY84	Keratin, type II cytoskeletal 71 OS=Homo sapiens OX=9606 GN=KRT71 PE=1 SV=3
Q14520	Hyaluronan-binding protein 2 OS=Homo sapiens OX=9606 GN=HABP2 PE=1 SV=1
Q07960	Rho GTPase-activating protein 1 OS=Homo sapiens OX=9606 GN=ARHGAP1 PE=1 SV=1
Q15404	Ras suppressor protein 1 OS=Homo sapiens OX=9606 GN=RSU1 PE=1 SV=3
P61981	14-3-3 protein gamma OS=Homo sapiens OX=9606 GN=YWHAG PE=1 SV=2
Q6KB66	Keratin, type II cytoskeletal 80 OS=Homo sapiens OX=9606 GN=KRT80 PE=1 SV=2
Q01813	ATP-dependent 6-phosphofructokinase, platelet type OS=Homo sapiens OX=9606 GN=PFKP PE=1 SV=2
O76011	Keratin, type I cuticular Ha4 OS=Homo sapiens OX=9606 GN=KRT34 PE=1 SV=2
P08311	Cathepsin G OS=Homo sapiens OX=9606 GN=CTSG PE=1 SV=2
P0DMV8	Heat shock 70 kDa protein 1A OS=Homo sapiens OX=9606 GN=HSPA1A PE=1 SV=1
P37837	Transaldolase OS=Homo sapiens OX=9606 GN=TALDO1 PE=1 SV=2
Q14019	Coactosin-like protein OS=Homo sapiens OX=9606 GN=COTL1 PE=1 SV=3
Q14525	Keratin, type I cuticular Ha3-II OS=Homo sapiens OX=9606 GN=KRT33B PE=1 SV=3
P78371	T-complex protein 1 subunit beta OS=Homo sapiens OX=9606 GN=CCT2 PE=1 SV=4
A0A0B4J1X5	Immunoglobulin heavy variable 3-74 OS=Homo sapiens OX=9606 GN=IGHV3-74 PE=3 SV=1
Q15084	Protein disulfide-isomerase A6 OS=Homo sapiens OX=9606 GN=PDIA6 PE=1 SV=1
P31150	Rab GDP dissociation inhibitor alpha OS=Homo sapiens OX=9606 GN=GDI1 PE=1 SV=2
P68371	Tubulin beta-4B chain OS=Homo sapiens OX=9606 GN=TUBB4B PE=1 SV=1
P04180	Phosphatidylcholine-sterol acyltransferase OS=Homo sapiens OX=9606 GN=LCAT PE=1 SV=1
P30740	Leukocyte elastase inhibitor OS=Homo sapiens OX=9606 GN=SERPINB1 PE=1 SV=1
P07359	Platelet glycoprotein Ib alpha chain OS=Homo sapiens OX=9606 GN=GP1BA PE=1 SV=2
Q9BR76	Coronin-1B OS=Homo sapiens OX=9606 GN=CORO1B PE=1 SV=1
P04075	Fructose-bisphosphate aldolase A OS=Homo sapiens OX=9606 GN=ALDOA PE=1 SV=2
P80748	Immunoglobulin lambda variable 3-21 OS=Homo sapiens OX=9606 GN=IGLV3-21 PE=1 SV=2
P55209	Nucleosome assembly protein 1-like 1 OS=Homo sapiens OX=9606 GN=NAP1L1 PE=1 SV=1
P07900	Heat shock protein HSP 90-alpha OS=Homo sapiens OX=9606 GN=HSP90AA1 PE=1 SV=5
P08729	Keratin, type II cytoskeletal 7 OS=Homo sapiens OX=9606 GN=KRT7 PE=1 SV=5
Q5JSH3	WD repeat-containing protein 44 OS=Homo sapiens OX=9606 GN=WDR44 PE=1 SV=1
P30041	Peroxiredoxin-6 OS=Homo sapiens OX=9606 GN=PRDX6 PE=1 SV=3
Q9NZN3	EH domain-containing protein 3 OS=Homo sapiens OX=9606 GN=EHD3 PE=1 SV=2
P06702	Protein S100-A9 OS=Homo sapiens OX=9606 GN=S100A9 PE=1 SV=1
P01780	Immunoglobulin heavy variable 3-7 OS=Homo sapiens OX=9606 GN=IGHV3-7 PE=1 SV=2
P01768	Immunoglobulin heavy variable 3-30 OS=Homo sapiens OX=9606 GN=IGHV3-30 PE=1 SV=2
O15143	Actin-related protein 2/3 complex subunit 1B OS=Homo sapiens OX=9606 GN=ARPC1B PE=1 SV=3
P48741	Putative heat shock 70 kDa protein 7 OS=Homo sapiens OX=9606 GN=HSPA7 PE=5 SV=2
P60660	Myosin light polypeptide 6 OS=Homo sapiens OX=9606 GN=MYL6 PE=1 SV=2
Q15942	Zyxin OS=Homo sapiens OX=9606 GN=ZYX PE=1 SV=1

P81605	Dermcidin OS=Homo sapiens OX=9606 GN=DCD PE=1 SV=2
P01880	Immunoglobulin heavy constant delta OS=Homo sapiens OX=9606 GN=IGHD PE=1 SV=3
P01591	Immunoglobulin J chain OS=Homo sapiens OX=9606 GN=JCHAIN PE=1 SV=4
P13224	Platelet glycoprotein Ib beta chain OS=Homo sapiens OX=9606 GN=GP1BB PE=1 SV=1
P48509	CD151 antigen OS=Homo sapiens OX=9606 GN=CD151 PE=1 SV=3
P0DOX7	Immunoglobulin kappa light chain OS=Homo sapiens OX=9606 PE=1 SV=1
P23284	Peptidyl-prolyl cis-trans isomerase B OS=Homo sapiens OX=9606 GN=PPIB PE=1 SV=2
P15153	Ras-related C3 botulinum toxin substrate 2 OS=Homo sapiens OX=9606 GN=RAC2 PE=1 SV=1
P05452	Tetranectin OS=Homo sapiens OX=9606 GN=CLEC3B PE=1 SV=3
P49747	Cartilage oligomeric matrix protein OS=Homo sapiens OX=9606 GN=COMP PE=1 SV=2
P61204	ADP-ribosylation factor 3 OS=Homo sapiens OX=9606 GN=ARF3 PE=1 SV=2
P60953	Cell division control protein 42 homolog OS=Homo sapiens OX=9606 GN=CDC42 PE=1 SV=2
P02652	Apolipoprotein A-II OS=Homo sapiens OX=9606 GN=APOA2 PE=1 SV=1
P01717	Immunoglobulin lambda variable 3-25 OS=Homo sapiens OX=9606 GN=IGLV3-25 PE=1 SV=2
Q92954	Proteoglycan 4 OS=Homo sapiens OX=9606 GN=PRG4 PE=1 SV=3
O43790	Keratin, type II cuticular Hb6 OS=Homo sapiens OX=9606 GN=KRT86 PE=1 SV=1
P18428	Lipopolysaccharide-binding protein OS=Homo sapiens OX=9606 GN=LBP PE=1 SV=3
P00915	Carbonic anhydrase 1 OS=Homo sapiens OX=9606 GN=CA1 PE=1 SV=2
P31151	Protein S100-A7 OS=Homo sapiens OX=9606 GN=S100A7 PE=1 SV=4
P24844	Myosin regulatory light polypeptide 9 OS=Homo sapiens OX=9606 GN=MYL9 PE=1 SV=4
O14950	Myosin regulatory light chain 12B OS=Homo sapiens OX=9606 GN=MYL12B PE=1 SV=2
P02655	Apolipoprotein C-II OS=Homo sapiens OX=9606 GN=APOC2 PE=1 SV=1
P78385	Keratin, type II cuticular Hb3 OS=Homo sapiens OX=9606 GN=KRT83 PE=1 SV=2
Q9H853	Putative tubulin-like protein alpha-4B OS=Homo sapiens OX=9606 GN=TUBA4B PE=5 SV=2
O76013	Keratin, type I cuticular Ha6 OS=Homo sapiens OX=9606 GN=KRT36 PE=2 SV=1
Q15323	Keratin, type I cuticular Ha1 OS=Homo sapiens OX=9606 GN=KRT31 PE=1 SV=3
Q15485	Ficolin-2 OS=Homo sapiens OX=9606 GN=FCN2 PE=1 SV=2
A6NCN2	Putative keratin-87 protein OS=Homo sapiens OX=9606 GN=KRT87P PE=5 SV=4
O15144	Actin-related protein 2/3 complex subunit 2 OS=Homo sapiens OX=9606 GN=ARPC2 PE=1 SV=1
P78386	Keratin, type II cuticular Hb5 OS=Homo sapiens OX=9606 GN=KRT85 PE=1 SV=1
Q9NSB4	Keratin, type II cuticular Hb2 OS=Homo sapiens OX=9606 GN=KRT82 PE=3 SV=3
P20930	Filaggrin OS=Homo sapiens OX=9606 GN=FLG PE=1 SV=3
Q9NZP8	Complement C1r subcomponent-like protein OS=Homo sapiens OX=9606 GN=C1RL PE=1 SV=2
A0A075B6K	Immunoglobulin lambda variable 3-10 OS=Homo sapiens OX=9606 GN=IGLV3-10 PE=3 SV=2
P19652	Alpha-1-acid glycoprotein 2 OS=Homo sapiens OX=9606 GN=ORM2 PE=1 SV=2

Supplementary Table 4. Protein profile obtained for MB.

Accesion number	Description
P60709	Actin, cytoplasmic 1 OS=Homo sapiens OX=9606 GN=ACTB PE=1 SV=1
P07437	Tubulin beta chain OS=Homo sapiens OX=9606 GN=TUBB PE=1 SV=2
P68133	Actin, alpha skeletal muscle OS=Homo sapiens OX=9606 GN=ACTA1 PE=1 SV=1
P01834	Immunoglobulin kappa constant OS=Homo sapiens OX=9606 GN=IGKC PE=1 SV=2

P04264	Keratin, type II cytoskeletal 1 OS=Homo sapiens OX=9606 GN=KRT1 PE=1 SV=6
P35527	Keratin, type I cytoskeletal 9 OS=Homo sapiens OX=9606 GN=KRT9 PE=1 SV=3
P02768	Serum albumin OS=Homo sapiens OX=9606 GN=ALB PE=1 SV=2
P68366	Tubulin alpha-4A chain OS=Homo sapiens OX=9606 GN=TUBA4A PE=1 SV=1
Q9HBI1	Beta-parvin OS=Homo sapiens OX=9606 GN=PARVB PE=1 SV=1
P01857	Immunoglobulin heavy constant gamma 1 OS=Homo sapiens OX=9606 GN=IGHG1 PE=1 SV=1
P35908	Keratin, type II cytoskeletal 2 epidermal OS=Homo sapiens OX=9606 GN=KRT2 PE=1 SV=2
P14618	Pyruvate kinase PKM OS=Homo sapiens OX=9606 GN=PKM PE=1 SV=4
P01008	Antithrombin-III OS=Homo sapiens OX=9606 GN=SERPINC1 PE=1 SV=1
P00450	Ceruloplasmin OS=Homo sapiens OX=9606 GN=CP PE=1 SV=1
P04406	Glyceraldehyde-3-phosphate dehydrogenase OS=Homo sapiens OX=9606 GN=GAPDH PE=1 SV=3
P13645	Keratin, type I cytoskeletal 10 OS=Homo sapiens OX=9606 GN=KRT10 PE=1 SV=6
P27918	Properdin OS=Homo sapiens OX=9606 GN=CFP PE=1 SV=2
P04217	Alpha-1B-glycoprotein OS=Homo sapiens OX=9606 GN=A1BG PE=1 SV=4
P27169	Serum paraoxonase/arylesterase 1 OS=Homo sapiens OX=9606 GN=PON1 PE=1 SV=3
P13647	Keratin, type II cytoskeletal 5 OS=Homo sapiens OX=9606 GN=KRT5 PE=1 SV=3
Q9H4B7	Tubulin beta-1 chain OS=Homo sapiens OX=9606 GN=TUBB1 PE=1 SV=1
Q96PD5	N-acetylmuramoyl-L-alanine amidase OS=Homo sapiens OX=9606 GN=PGLYRP2 PE=1 SV=1
P00558	Phosphoglycerate kinase 1 OS=Homo sapiens OX=9606 GN=PGK1 PE=1 SV=3
P68363	Tubulin alpha-1B chain OS=Homo sapiens OX=9606 GN=TUBA1B PE=1 SV=1
Q96IY4	Carboxypeptidase B2 OS=Homo sapiens OX=9606 GN=CPB2 PE=1 SV=2
P00734	Prothrombin OS=Homo sapiens OX=9606 GN=F2 PE=1 SV=2
Q9Y490	Talin-1 OS=Homo sapiens OX=9606 GN=TLN1 PE=1 SV=3
P08603	Complement factor H OS=Homo sapiens OX=9606 GN=CFH PE=1 SV=4
P07360	Complement component C8 gamma chain OS=Homo sapiens OX=9606 GN=C8G PE=1 SV=3
Q562R1	Beta-actin-like protein 2 OS=Homo sapiens OX=9606 GN=ACTBL2 PE=1 SV=2
P52907	F-actin-capping protein subunit alpha-1 OS=Homo sapiens OX=9606 GN=CAPZA1 PE=1 SV=3
P07225	Vitamin K-dependent protein S OS=Homo sapiens OX=9606 GN=PROS1 PE=1 SV=1
P02675	Fibrinogen beta chain OS=Homo sapiens OX=9606 GN=FGB PE=1 SV=2
P30101	Protein disulfide-isomerase A3 OS=Homo sapiens OX=9606 GN=PDIA3 PE=1 SV=4
P06396	Gelsolin OS=Homo sapiens OX=9606 GN=GSN PE=1 SV=1
P01031	Complement C5 OS=Homo sapiens OX=9606 GN=C5 PE=1 SV=4
P02790	Hemopexin OS=Homo sapiens OX=9606 GN=HPX PE=1 SV=2
P08514	Integrin alpha-IIb OS=Homo sapiens OX=9606 GN=ITGA2B PE=1 SV=3
Q14624	Inter-alpha-trypsin inhibitor heavy chain H4 OS=Homo sapiens OX=9606 GN=ITIH4 PE=1 SV=4
P02760	Protein AMBP OS=Homo sapiens OX=9606 GN=AMBP PE=1 SV=1
P48740	Mannan-binding lectin serine protease 1 OS=Homo sapiens OX=9606 GN=MASP1 PE=1 SV=3
P01009	Alpha-1-antitrypsin OS=Homo sapiens OX=9606 GN=SERPINA1 PE=1 SV=3
P01859	Immunoglobulin heavy constant gamma 2 OS=Homo sapiens OX=9606 GN=IGHG2 PE=1 SV=2
P00738	Haptoglobin OS=Homo sapiens OX=9606 GN=HP PE=1 SV=1
P35858	Insulin-like growth factor-binding protein complex acid labile subunit OS=Homo sapiens OX=9606 GN=IGFALS PE=1 SV=1
P40197	Platelet glycoprotein V OS=Homo sapiens OX=9606 GN=GP5 PE=1 SV=1
P08567	Pleckstrin OS=Homo sapiens OX=9606 GN=PLEK PE=1 SV=3

O75083	WD repeat-containing protein 1 OS=Homo sapiens OX=9606 GN=WDR1 PE=1 SV=4
P01023	Alpha-2-macroglobulin OS=Homo sapiens OX=9606 GN=A2M PE=1 SV=3
Q9ULV4	Coronin-1C OS=Homo sapiens OX=9606 GN=CORO1C PE=1 SV=1
Q06033	Inter-alpha-trypsin inhibitor heavy chain H3 OS=Homo sapiens OX=9606 GN=ITIH3 PE=1 SV=2
P04004	Vitronectin OS=Homo sapiens OX=9606 GN=VTN PE=1 SV=1
P02746	Complement C1q subcomponent subunit B OS=Homo sapiens OX=9606 GN=C1QB PE=1 SV=3
P51884	Lumican OS=Homo sapiens OX=9606 GN=LUM PE=1 SV=2
P07358	Complement component C8 beta chain OS=Homo sapiens OX=9606 GN=C8B PE=1 SV=3
P02749	Beta-2-glycoprotein 1 OS=Homo sapiens OX=9606 GN=APOH PE=1 SV=3
P02774	Vitamin D-binding protein OS=Homo sapiens OX=9606 GN=GC PE=1 SV=2
Q86UX7	Fermitin family homolog 3 OS=Homo sapiens OX=9606 GN=FERMT3 PE=1 SV=1
P00736	Complement C1r subcomponent OS=Homo sapiens OX=9606 GN=C1R PE=1 SV=2
P21333	Filamin-A OS=Homo sapiens OX=9606 GN=FLNA PE=1 SV=4
P01871	Immunoglobulin heavy constant mu OS=Homo sapiens OX=9606 GN=IGHM PE=1 SV=4
P00742	Coagulation factor X OS=Homo sapiens OX=9606 GN=F10 PE=1 SV=2
P09871	Complement C1s subcomponent OS=Homo sapiens OX=9606 GN=C1S PE=1 SV=1
P35579	Myosin-9 OS=Homo sapiens OX=9606 GN=MYH9 PE=1 SV=4
Q15166	Serum paraoxonase/lactonase 3 OS=Homo sapiens OX=9606 GN=PON3 PE=1 SV=3
P12814	Alpha-actinin-1 OS=Homo sapiens OX=9606 GN=ACTN1 PE=1 SV=2
P02751	Fibronectin OS=Homo sapiens OX=9606 GN=FN1 PE=1 SV=5
P20742	Pregnancy zone protein OS=Homo sapiens OX=9606 GN=PZP PE=1 SV=4
P05155	Plasma protease C1 inhibitor OS=Homo sapiens OX=9606 GN=SERPING1 PE=1 SV=2
P11142	Heat shock cognate 71 kDa protein OS=Homo sapiens OX=9606 GN=HSPA8 PE=1 SV=1
P02765	Alpha-2-HS-glycoprotein OS=Homo sapiens OX=9606 GN=AHSG PE=1 SV=2
P43652	Afamin OS=Homo sapiens OX=9606 GN=AFM PE=1 SV=1
P11021	Endoplasmic reticulum chaperone BiP OS=Homo sapiens OX=9606 GN=HSPA5 PE=1 SV=2
P19827	Inter-alpha-trypsin inhibitor heavy chain H1 OS=Homo sapiens OX=9606 GN=ITIH1 PE=1 SV=3
P02748	Complement component C9 OS=Homo sapiens OX=9606 GN=C9 PE=1 SV=2
P29622	Kallistatin OS=Homo sapiens OX=9606 GN=SERPINA4 PE=1 SV=3
P06733	Alpha-enolase OS=Homo sapiens OX=9606 GN=ENO1 PE=1 SV=2
P02671	Fibrinogen alpha chain OS=Homo sapiens OX=9606 GN=FGA PE=1 SV=2
P00751	Complement factor B OS=Homo sapiens OX=9606 GN=CFB PE=1 SV=2
P19823	Inter-alpha-trypsin inhibitor heavy chain H2 OS=Homo sapiens OX=9606 GN=ITIH2 PE=1 SV=2
P36955	Pigment epithelium-derived factor OS=Homo sapiens OX=9606 GN=SERPINF1 PE=1 SV=4
P02649	Apolipoprotein E OS=Homo sapiens OX=9606 GN=APOE PE=1 SV=1
P07996	Thrombospondin-1 OS=Homo sapiens OX=9606 GN=THBS1 PE=1 SV=2
P02679	Fibrinogen gamma chain OS=Homo sapiens OX=9606 GN=FGG PE=1 SV=3
P02747	Complement C1q subcomponent subunit C OS=Homo sapiens OX=9606 GN=C1QC PE=1 SV=3
P18206	Vinculin OS=Homo sapiens OX=9606 GN=VCL PE=1 SV=4
P01861	Immunoglobulin heavy constant gamma 4 OS=Homo sapiens OX=9606 GN=IGHG4 PE=1 SV=1
Q13418	Integrin-linked protein kinase OS=Homo sapiens OX=9606 GN=ILK PE=1 SV=2
P01024	Complement C3 OS=Homo sapiens OX=9606 GN=C3 PE=1 SV=2
P04003	C4b-binding protein alpha chain OS=Homo sapiens OX=9606 GN=C4BPA PE=1 SV=2
P01011	Alpha-1-antichymotrypsin OS=Homo sapiens OX=9606 GN=SERPINA3 PE=1 SV=2

P10643	Complement component C7 OS=Homo sapiens OX=9606 GN=C7 PE=1 SV=2
P14625	Endoplasmin OS=Homo sapiens OX=9606 GN=HSP90B1 PE=1 SV=1
P00747	Plasminogen OS=Homo sapiens OX=9606 GN=PLG PE=1 SV=2
P31146	Coronin-1A OS=Homo sapiens OX=9606 GN=CORO1A PE=1 SV=4
P08697	Alpha-2-antiplasmin OS=Homo sapiens OX=9606 GN=SERPINF2 PE=1 SV=3
P01876	Immunoglobulin heavy constant alpha 1 OS=Homo sapiens OX=9606 GN=IGHA1 PE=1 SV=2
P13671	Complement component C6 OS=Homo sapiens OX=9606 GN=C6 PE=1 SV=3
P07737	Profilin-1 OS=Homo sapiens OX=9606 GN=PFN1 PE=1 SV=2
P10909	Clusterin OS=Homo sapiens OX=9606 GN=CLU PE=1 SV=1
P26038	Moesin OS=Homo sapiens OX=9606 GN=MSN PE=1 SV=3
P52566	Rho GDP-dissociation inhibitor 2 OS=Homo sapiens OX=9606 GN=ARHGDIB PE=1 SV=3
P06681	Complement C2 OS=Homo sapiens OX=9606 GN=C2 PE=1 SV=2
P22792	Carboxypeptidase N subunit 2 OS=Homo sapiens OX=9606 GN=CPN2 PE=1 SV=3
P50552	Vasodilator-stimulated phosphoprotein OS=Homo sapiens OX=9606 GN=VASP PE=1 SV=3
P03952	Plasma kallikrein OS=Homo sapiens OX=9606 GN=KLKB1 PE=1 SV=1
P07357	Complement component C8 alpha chain OS=Homo sapiens OX=9606 GN=C8A PE=1 SV=2
P00739	Haptoglobin-related protein OS=Homo sapiens OX=9606 GN=HPR PE=2 SV=2
P37802	Transgelin-2 OS=Homo sapiens OX=9606 GN=TAGLN2 PE=1 SV=3
P05106	Integrin beta-3 OS=Homo sapiens OX=9606 GN=ITGB3 PE=1 SV=2
P15169	Carboxypeptidase N catalytic chain OS=Homo sapiens OX=9606 GN=CPN1 PE=1 SV=1
Q04917	14-3-3 protein eta OS=Homo sapiens OX=9606 GN=YWHAH PE=1 SV=4
P27105	Erythrocyte band 7 integral membrane protein OS=Homo sapiens OX=9606 GN=STOM PE=1 SV=3
P12931	Proto-oncogene tyrosine-protein kinase Src OS=Homo sapiens OX=9606 GN=SRC PE=1 SV=3
P63104	14-3-3 protein zeta/delta OS=Homo sapiens OX=9606 GN=YWHAZ PE=1 SV=1
P06727	Apolipoprotein A-IV OS=Homo sapiens OX=9606 GN=APOA4 PE=1 SV=3
P05546	Heparin cofactor 2 OS=Homo sapiens OX=9606 GN=SERPIND1 PE=1 SV=3
P67936	Tropomyosin alpha-4 chain OS=Homo sapiens OX=9606 GN=TPM4 PE=1 SV=3
Q01518	Adenylyl cyclase-associated protein 1 OS=Homo sapiens OX=9606 GN=CAP1 PE=1 SV=5
O43866	CD5 antigen-like OS=Homo sapiens OX=9606 GN=CD5L PE=1 SV=1
P06744	Glucose-6-phosphate isomerase OS=Homo sapiens OX=9606 GN=GPI PE=1 SV=4
P21926	CD9 antigen OS=Homo sapiens OX=9606 GN=CD9 PE=1 SV=4
P04196	Histidine-rich glycoprotein OS=Homo sapiens OX=9606 GN=HRG PE=1 SV=1
Q9UBW5	Bridging integrator 2 OS=Homo sapiens OX=9606 GN=BIN2 PE=1 SV=3
A0A0B4J1V0	Immunoglobulin heavy variable 3-15 OS=Homo sapiens OX=9606 GN=IGHV3-15 PE=3 SV=1
P02750	Leucine-rich alpha-2-glycoprotein OS=Homo sapiens OX=9606 GN=LRG1 PE=1 SV=2
Q16610	Extracellular matrix protein 1 OS=Homo sapiens OX=9606 GN=ECM1 PE=1 SV=2
P23142	Fibulin-1 OS=Homo sapiens OX=9606 GN=FBLN1 PE=1 SV=4
O00299	Chloride intracellular channel protein 1 OS=Homo sapiens OX=9606 GN=CLIC1 PE=1 SV=4
P01042	Kininogen-1 OS=Homo sapiens OX=9606 GN=KNG1 PE=1 SV=2
P04275	von Willebrand factor OS=Homo sapiens OX=9606 GN=VWF PE=1 SV=4
P69905	Hemoglobin subunit alpha OS=Homo sapiens OX=9606 GN=HBA1 PE=1 SV=2
P0C0L5	Complement C4-B OS=Homo sapiens OX=9606 GN=C4B PE=1 SV=2
P04114	Apolipoprotein B-100 OS=Homo sapiens OX=9606 GN=APOB PE=1 SV=2
P0C0L4	Complement C4-A OS=Homo sapiens OX=9606 GN=C4A PE=1 SV=2

P01860	Immunoglobulin heavy constant gamma 3 OS=Homo sapiens OX=9606 GN=IGHG3 PE=1 SV=2
P08185	Corticosteroid-binding globulin OS=Homo sapiens OX=9606 GN=SERPINA6 PE=1 SV=1
P08246	Neutrophil elastase OS=Homo sapiens OX=9606 GN=ELANE PE=1 SV=1
P00488	Coagulation factor XIII A chain OS=Homo sapiens OX=9606 GN=F13A1 PE=1 SV=4
P02745	Complement C1q subcomponent subunit A OS=Homo sapiens OX=9606 GN=C1QA PE=1 SV=2
P08571	Monocyte differentiation antigen CD14 OS=Homo sapiens OX=9606 GN=CD14 PE=1 SV=2
O14791	Apolipoprotein L1 OS=Homo sapiens OX=9606 GN=APOL1 PE=1 SV=5
P60174	Triosephosphate isomerase OS=Homo sapiens OX=9606 GN=TPI1 PE=1 SV=3
P12259	Coagulation factor V OS=Homo sapiens OX=9606 GN=F5 PE=1 SV=4
P00740	Coagulation factor IX OS=Homo sapiens OX=9606 GN=F9 PE=1 SV=2
Q13201	Multimerin-1 OS=Homo sapiens OX=9606 GN=MMRN1 PE=1 SV=3
Q2TV78	Putative macrophage stimulating 1-like protein OS=Homo sapiens OX=9606 GN=MST1L PE=2 SV=2
P00918	Carbonic anhydrase 2 OS=Homo sapiens OX=9606 GN=CA2 PE=1 SV=2
O95810	Caveolae-associated protein 2 OS=Homo sapiens OX=9606 GN=CAVIN2 PE=1 SV=3
P0DOX8	Immunoglobulin lambda-1 light chain OS=Homo sapiens OX=9606 PE=1 SV=1
P48059	LIM and senescent cell antigen-like-containing domain protein 1 OS=Homo sapiens OX=9606 GN=LIMS1 PE=1 SV=4
P61224	Ras-related protein Rap-1b OS=Homo sapiens OX=9606 GN=RAP1B PE=1 SV=1
P59665	Neutrophil defensin 1 OS=Homo sapiens OX=9606 GN=DEFA1 PE=1 SV=1
P0DOY3	Immunoglobulin lambda constant 3 OS=Homo sapiens OX=9606 GN=IGLC3 PE=1 SV=1
O75636	Ficolin-3 OS=Homo sapiens OX=9606 GN=FCN3 PE=1 SV=2
P02647	Apolipoprotein A-I OS=Homo sapiens OX=9606 GN=APOA1 PE=1 SV=1
P62937	Peptidyl-prolyl cis-trans isomerase A OS=Homo sapiens OX=9606 GN=PPIA PE=1 SV=2
P68871	Hemoglobin subunit beta OS=Homo sapiens OX=9606 GN=HBB PE=1 SV=2
P35542	Serum amyloid A-4 protein OS=Homo sapiens OX=9606 GN=SAA4 PE=1 SV=2
P61158	Actin-related protein 3 OS=Homo sapiens OX=9606 GN=ACTR3 PE=1 SV=3
P20851	C4b-binding protein beta chain OS=Homo sapiens OX=9606 GN=C4BPB PE=1 SV=1
P33908	Mannosyl-oligosaccharide 1,2-alpha-mannosidase IA OS=Homo sapiens OX=9606 GN=MAN1A1 PE=1 SV=3
P27797	Calreticulin OS=Homo sapiens OX=9606 GN=CALR PE=1 SV=1
P23528	Cofilin-1 OS=Homo sapiens OX=9606 GN=CFL1 PE=1 SV=3
P62491	Ras-related protein Rab-11A OS=Homo sapiens OX=9606 GN=RAB11A PE=1 SV=3
P08519	Apolipoprotein(a) OS=Homo sapiens OX=9606 GN=LPA PE=1 SV=1
P61160	Actin-related protein 2 OS=Homo sapiens OX=9606 GN=ACTR2 PE=1 SV=1
P78417	Glutathione S-transferase omega-1 OS=Homo sapiens OX=9606 GN=GSTO1 PE=1 SV=2
P49908	Selenoprotein P OS=Homo sapiens OX=9606 GN=SELENOP PE=1 SV=3
P31946	14-3-3 protein beta/alpha OS=Homo sapiens OX=9606 GN=YWHAB PE=1 SV=3
P11413	Glucose-6-phosphate 1-dehydrogenase OS=Homo sapiens OX=9606 GN=G6PD PE=1 SV=4
P02766	Transthyretin OS=Homo sapiens OX=9606 GN=TTR PE=1 SV=1
O95445	Apolipoprotein M OS=Homo sapiens OX=9606 GN=APOM PE=1 SV=2
P04278	Sex hormone-binding globulin OS=Homo sapiens OX=9606 GN=SHBG PE=1 SV=2
P04259	Keratin, type II cytoskeletal 6B OS=Homo sapiens OX=9606 GN=KRT6B PE=1 SV=5
P08779	Keratin, type I cytoskeletal 16 OS=Homo sapiens OX=9606 GN=KRT16 PE=1 SV=4

A0A075B6I9	Immunoglobulin lambda variable 7-46 OS=Homo sapiens OX=9606 GN=IGLV7-46 PE=3 SV=4
P63261	Actin, cytoplasmic 2 OS=Homo sapiens OX=9606 GN=ACTG1 PE=1 SV=1
P61026	Ras-related protein Rab-10 OS=Homo sapiens OX=9606 GN=RAB10 PE=1 SV=1
P02533	Keratin, type I cytoskeletal 14 OS=Homo sapiens OX=9606 GN=KRT14 PE=1 SV=4
P0DOX6	Immunoglobulin mu heavy chain OS=Homo sapiens OX=9606 PE=1 SV=2
Q04695	Keratin, type I cytoskeletal 17 OS=Homo sapiens OX=9606 GN=KRT17 PE=1 SV=2
Q5D862	Filaggrin-2 OS=Homo sapiens OX=9606 GN=FLG2 PE=1 SV=1
P01019	Angiotensinogen OS=Homo sapiens OX=9606 GN=AGT PE=1 SV=1
P0DOX2	Immunoglobulin alpha-2 heavy chain OS=Homo sapiens OX=9606 PE=1 SV=2
Q9UK55	Protein Z-dependent protease inhibitor OS=Homo sapiens OX=9606 GN=SERPINA10 PE=1 SV=1
Q86YZ3	Hornerin OS=Homo sapiens OX=9606 GN=HRNR PE=1 SV=2
Q5T749	Keratinocyte proline-rich protein OS=Homo sapiens OX=9606 GN=KPRP PE=1 SV=1
P29508	Serpin B3 OS=Homo sapiens OX=9606 GN=SERPINB3 PE=1 SV=2
P05109	Protein S100-A8 OS=Homo sapiens OX=9606 GN=S100A8 PE=1 SV=1
P02538	Keratin, type II cytoskeletal 6A OS=Homo sapiens OX=9606 GN=KRT6A PE=1 SV=3
Q14766	Latent-transforming growth factor beta-binding protein 1 OS=Homo sapiens OX=9606 GN=LTBP1 PE=1 SV=4
P02787	Serotransferrin OS=Homo sapiens OX=9606 GN=TF PE=1 SV=3
P01619	Immunoglobulin kappa variable 3-20 OS=Homo sapiens OX=9606 GN=IGKV3-20 PE=1 SV=2
P14923	Junction plakoglobin OS=Homo sapiens OX=9606 GN=JUP PE=1 SV=3
P15924	Desmoplakin OS=Homo sapiens OX=9606 GN=DSP PE=1 SV=3
Q3SY84	Keratin, type II cytoskeletal 71 OS=Homo sapiens OX=9606 GN=KRT71 PE=1 SV=3
Q14520	Hyaluronan-binding protein 2 OS=Homo sapiens OX=9606 GN=HABP2 PE=1 SV=1
Q07960	Rho GTPase-activating protein 1 OS=Homo sapiens OX=9606 GN=ARHGAP1 PE=1 SV=1
Q15404	Ras suppressor protein 1 OS=Homo sapiens OX=9606 GN=RSU1 PE=1 SV=3
P61981	14-3-3 protein gamma OS=Homo sapiens OX=9606 GN=YWHAG PE=1 SV=2
Q6KB66	Keratin, type II cytoskeletal 80 OS=Homo sapiens OX=9606 GN=KRT80 PE=1 SV=2
Q01813	ATP-dependent 6-phosphofructokinase, platelet type OS=Homo sapiens OX=9606 GN=PFKP PE=1 SV=2
O76011	Keratin, type I cuticular Ha4 OS=Homo sapiens OX=9606 GN=KRT34 PE=1 SV=2
P08311	Cathepsin G OS=Homo sapiens OX=9606 GN=CTSG PE=1 SV=2
P0DMV8	Heat shock 70 kDa protein 1A OS=Homo sapiens OX=9606 GN=HSPA1A PE=1 SV=1
P37837	Transaldolase OS=Homo sapiens OX=9606 GN=TALDO1 PE=1 SV=2
Q14019	Coactosin-like protein OS=Homo sapiens OX=9606 GN=COTL1 PE=1 SV=3
Q14525	Keratin, type I cuticular Ha3-II OS=Homo sapiens OX=9606 GN=KRT33B PE=1 SV=3
P78371	T-complex protein 1 subunit beta OS=Homo sapiens OX=9606 GN=CCT2 PE=1 SV=4
A0A0B4J1X5	Immunoglobulin heavy variable 3-74 OS=Homo sapiens OX=9606 GN=IGHV3-74 PE=3 SV=1
Q15084	Protein disulfide-isomerase A6 OS=Homo sapiens OX=9606 GN=PDIA6 PE=1 SV=1
P31150	Rab GDP dissociation inhibitor alpha OS=Homo sapiens OX=9606 GN=GDI1 PE=1 SV=2
P68371	Tubulin beta-4B chain OS=Homo sapiens OX=9606 GN=TUBB4B PE=1 SV=1
P04180	Phosphatidylcholine-sterol acyltransferase OS=Homo sapiens OX=9606 GN=LCAT PE=1 SV=1
P30740	Leukocyte elastase inhibitor OS=Homo sapiens OX=9606 GN=SERPINB1 PE=1 SV=1
P07359	Platelet glycoprotein Ib alpha chain OS=Homo sapiens OX=9606 GN=GP1BA PE=1 SV=2
Q9BR76	Coronin-1B OS=Homo sapiens OX=9606 GN=CORO1B PE=1 SV=1

P04075	Fructose-bisphosphate aldolase A OS=Homo sapiens OX=9606 GN=ALDOA PE=1 SV=2
P80748	Immunoglobulin lambda variable 3-21 OS=Homo sapiens OX=9606 GN=IGLV3-21 PE=1 SV=2
P55209	Nucleosome assembly protein 1-like 1 OS=Homo sapiens OX=9606 GN=NAP1L1 PE=1 SV=1
P07900	Heat shock protein HSP 90-alpha OS=Homo sapiens OX=9606 GN=HSP90AA1 PE=1 SV=5
P08729	Keratin, type II cytoskeletal 7 OS=Homo sapiens OX=9606 GN=KRT7 PE=1 SV=5
Q5JSH3	WD repeat-containing protein 44 OS=Homo sapiens OX=9606 GN=WDR44 PE=1 SV=1
P30041	Peroxiredoxin-6 OS=Homo sapiens OX=9606 GN=PRDX6 PE=1 SV=3
Q9NZN3	EH domain-containing protein 3 OS=Homo sapiens OX=9606 GN=EHD3 PE=1 SV=2
P06702	Protein S100-A9 OS=Homo sapiens OX=9606 GN=S100A9 PE=1 SV=1
P01780	Immunoglobulin heavy variable 3-7 OS=Homo sapiens OX=9606 GN=IGHV3-7 PE=1 SV=2
P01768	Immunoglobulin heavy variable 3-30 OS=Homo sapiens OX=9606 GN=IGHV3-30 PE=1 SV=2
O15143	Actin-related protein 2/3 complex subunit 1B OS=Homo sapiens OX=9606 GN=ARPC1B PE=1 SV=3
P48741	Putative heat shock 70 kDa protein 7 OS=Homo sapiens OX=9606 GN=HSPA7 PE=5 SV=2
P60660	Myosin light polypeptide 6 OS=Homo sapiens OX=9606 GN=MYL6 PE=1 SV=2
Q15942	Zyxin OS=Homo sapiens OX=9606 GN=ZYX PE=1 SV=1
P81605	Dermcidin OS=Homo sapiens OX=9606 GN=DCD PE=1 SV=2
P01880	Immunoglobulin heavy constant delta OS=Homo sapiens OX=9606 GN=IGHD PE=1 SV=3
P01591	Immunoglobulin J chain OS=Homo sapiens OX=9606 GN=JCHAIN PE=1 SV=4
P13224	Platelet glycoprotein Ib beta chain OS=Homo sapiens OX=9606 GN=GP1BB PE=1 SV=1
P48509	CD151 antigen OS=Homo sapiens OX=9606 GN=CD151 PE=1 SV=3
P0DOX7	Immunoglobulin kappa light chain OS=Homo sapiens OX=9606 PE=1 SV=1
P23284	Peptidyl-prolyl cis-trans isomerase B OS=Homo sapiens OX=9606 GN=PPIB PE=1 SV=2
P15153	Ras-related C3 botulinum toxin substrate 2 OS=Homo sapiens OX=9606 GN=RAC2 PE=1 SV=1
P05452	Tetranectin OS=Homo sapiens OX=9606 GN=CLEC3B PE=1 SV=3
P49747	Cartilage oligomeric matrix protein OS=Homo sapiens OX=9606 GN=COMP PE=1 SV=2
P61204	ADP-ribosylation factor 3 OS=Homo sapiens OX=9606 GN=ARF3 PE=1 SV=2
P60953	Cell division control protein 42 homolog OS=Homo sapiens OX=9606 GN=CDC42 PE=1 SV=2
P02652	Apolipoprotein A-II OS=Homo sapiens OX=9606 GN=APOA2 PE=1 SV=1
P01717	Immunoglobulin lambda variable 3-25 OS=Homo sapiens OX=9606 GN=IGLV3-25 PE=1 SV=2
Q92954	Proteoglycan 4 OS=Homo sapiens OX=9606 GN=PRG4 PE=1 SV=3
O43790	Keratin, type II cuticular Hb6 OS=Homo sapiens OX=9606 GN=KRT86 PE=1 SV=1
P18428	Lipopolysaccharide-binding protein OS=Homo sapiens OX=9606 GN=LBP PE=1 SV=3
P00915	Carbonic anhydrase 1 OS=Homo sapiens OX=9606 GN=CA1 PE=1 SV=2
P24844	Myosin regulatory light polypeptide 9 OS=Homo sapiens OX=9606 GN=MYL9 PE=1 SV=4
O14950	Myosin regulatory light chain 12B OS=Homo sapiens OX=9606 GN=MYL12B PE=1 SV=2
P02655	Apolipoprotein C-II OS=Homo sapiens OX=9606 GN=APOC2 PE=1 SV=1
P78385	Keratin, type II cuticular Hb3 OS=Homo sapiens OX=9606 GN=KRT83 PE=1 SV=2
Q9H853	Putative tubulin-like protein alpha-4B OS=Homo sapiens OX=9606 GN=TUBA4B PE=5 SV=2
P01709	Immunoglobulin lambda variable 2-8 OS=Homo sapiens OX=9606 GN=IGLV2-8 PE=1 SV=2
Q92764	Keratin, type I cuticular Ha5 OS=Homo sapiens OX=9606 GN=KRT35 PE=2 SV=5

Supplementary Table 5. Associated gene names of the common proteins identified in other published studies according to the methodology analyzed.

SDS-PAGE	OSP	MB
A2M	A2M	A2M
ACTA1	ACTA1	ACTA1
ACTB	ACTB	ACTB
ACTBL2	ACTBL2	ACTBL2
ACTG1	ACTG1	ACTG1
ACTN1	ACTN1	ACTN1
ACTR2	ACTR2	ACTR2
ACTR3	ACTR3	ACTR3
ALB	ALB	ALB
ALDOA	ALDOA	ALDOA
APOA1	APOA1	APOA1
APOB	APOB	APOB
APOE	APOE	APOE
ARF3	ARF3	ARF3
ARHGAP1	ARHGAP1	ARHGAP1
ARHGDIB	ARHGDIB	ARHGDIB
ARPC1B	ARPC1B	ARPC1B
ARPC2	ARPC2	-
BIN2	BIN2	BIN2
C3	C3	C3
CA1	CA1	CA1
CA2	CA2	CA2
CALR	CALR	CALR
CAP1	CAP1	CAP1
CAPZA1	CAPZA1	CAPZA1
CAVIN2	CAVIN2	CAVIN2
CCT2	CCT2	CCT2
CD9	CD9	CD9
CDC42	CDC42	CDC42
CFL1	CFL1	CFL1
CLIC1	CLIC1	CLIC1
CLU	CLU	CLU
CORO1A	CORO1A	CORO1A
CORO1B	CORO1B	CORO1B
CORO1C	CORO1C	CORO1C
COTL1	COTL1	COTL1
CTSG	CTSG	CTSG
DCD	DCD	DCD
DSP	DSP	DSP

EHD3	EHD3	EHD3
ELANE	ELANE	ELANE
ENO1	ENO1	ENO1
F13A1	F13A1	F13A1
F5	F5	F5
FBLN1	FBLN1	FBLN1
FERMT3	FERMT3	FERMT3
FGA	FGA	FGA
FGB	FGB	FGB
FGG	FGG	FGG
FLG2	FLG2	FLG2
FLNA	FLNA	FLNA
FN1	FN1	FN1
G6PD	G6PD	G6PD
GAPDH	GAPDH	GAPDH
GDI1	GDI1	GDI1
GP1BA	GP1BA	GP1BA
GP1BB	GP1BB	GP1BB
GP5	GP5	GP5
GPI	GPI	GPI
GSN	GSN	GSN
GSTO1	GSTO1	GSTO1
HBA1	HBA1	HBA1
HBB	HBB	HBB
HRG	HRG	HRG
HRNR	HRNR	HRNR
HSP90AA1	HSP90AA1	HSP90AA1
HSP90AB1	-	-
HSP90B1	HSP90B1	HSP90B1
HSPA1A	HSPA1A	HSPA1A
HSPA5	HSPA5	HSPA5
HSPA8	HSPA8	HSPA8
ILK	ILK	ILK
ITGA2B	ITGA2B	ITGA2B
ITGB3	ITGB3	ITGB3
KRT1	KRT1	KRT1
KRT10	KRT10	KRT10
KRT14	KRT14	KRT14
KRT16	KRT16	KRT16
KRT2	KRT2	KRT2
KRT33B	KRT33B	KRT33B
KRT5	KRT5	KRT5
KRT6A	KRT6A	KRT6A
KRT9	KRT9	KRT9

LIMS1	LIMS1	LIMS1
LTBP1	LTBP1	LTBP1
MMRN1	MMRN1	MMRN1
MSN	MSN	MSN
MYH9	MYH9	MYH9
-	MYL12B	MYL12B
MYL6	MYL6	MYL6
-	MYL9	MYL9
NAP1L1	NAP1L1	NAP1L1
PARVB	PARVB	PARVB
PDIA3	PDIA3	PDIA3
PDIA6	PDIA6	PDIA6
PFKP	PFKP	PFKP
PFN1	PFN1	PFN1
PGK1	PGK1	PGK1
PKM	PKM	PKM
PLEK	PLEK	PLEK
PLG	PLG	PLG
PPIA	PPIA	PPIA
PPIB	PPIB	PPIB
PRDX6	PRDX6	PRDX6
PROS1	PROS1	PROS1
RAB10	RAB10	RAB10
RAC2	RAC2	RAC2
RAP1B	RAP1B	RAP1B
RSU1	RSU1	RSU1
S100A8	S100A8	S100A8
S100A9	S100A9	S100A9
SEPTIN7	-	-
SERPINA1	SERPINA1	SERPINA1
SERPINB1	SERPINB1	SERPINB1
SRC	SRC	SRC
STOM	STOM	STOM
TAGLN2	TAGLN2	TAGLN2
TALDO1	TALDO1	TALDO1
TF	TF	TF
THBS1	THBS1	THBS1
TLN1	TLN1	TLN1
TPI1	TPI1	TPI1
TPM4	TPM4	TPM4
TTR	TTR	TTR
TUBA1B	TUBA1B	TUBA1B
TUBA4A	TUBA4A	TUBA4A
TUBB	TUBB	TUBB

TUBB1	TUBB1	TUBB1
VASP	VASP	VASP
VCL	VCL	VCL
VTN	VTN	VTN
VWF	VWF	VWF
WDR1	WDR1	WDR1
WDR44	WDR44	WDR44
YWHAB	YWHAB	YWHAB
YWHAG	YWHAG	YWHAG
YWHAH	YWHAH	YWHAH
YWHAQ	-	-
YWHAZ	YWHAZ	YWHAZ
ZYX	ZYX	ZYX

Supplementary Table 6. Associated gene name of the unique proteins only identified in our study according to the methodology analyzed.

SDS-PAGE	OSP	MB
A1BG	A1BG	A1BG
AFM	AFM	AFM
AGT	AGT	AGT
AHSG	AHSG	AHSG
AMBP	AMBP	AMBP
APOA2	APOA2	APOA2
APOA4	APOA4	APOA4
-	APOC2	APOC2
APOH	APOH	APOH
APOL1	APOL1	APOL1
APOM	APOM	APOM
C1QA	C1QA	C1QA
C1QB	C1QB	C1QB
C1QC	C1QC	C1QC
C1R	C1R	C1R
C1RL	C1RL	-
C1S	C1S	C1S
C2	C2	C2
C4A	C4A	C4A
C4B	C4B	C4B
C4BPA	C4BPA	C4BPA
C4BPB	C4BPB	C4BPB
C5	C5	C5

C6	C6	C6
C7	C7	C7
C8A	C8A	C8A
C8B	C8B	C8B
C8G	C8G	C8G
C9	C9	C9
CD14	CD14	CD14
CD151	CD151	CD151
CD5L	CD5L	CD5L
CFB	CFB	CFB
CFH	CFH	CFH
CFP	CFP	CFP
CLEC3B	CLEC3B	CLEC3B
COMP	COMP	COMP
CP	CP	CP
CPB2	CPB2	CPB2
CPN1	CPN1	CPN1
CPN2	CPN2	CPN2
DEFA1	DEFA1	DEFA1
ECM1	ECM1	ECM1
F10	F10	F10
F2	F2	F2
F9	F9	F9
FCN2	FCN2	-
FCN3	FCN3	FCN3
FLG	-	-
GC	GC	GC
HABP2	HABP2	HABP2
HP	HP	HP
HPR	HPR	HPR
HPX	HPX	HPX
HSPA7	HSPA7	HSPA7
IGA	IGA	IGA
IGFALS	IGFALS	IGFALS
IGHA1	IGHA1	IGHA1
IGHD	IGHD	IGHD
IGHG1	IGHG1	IGHG1
IGHG2	IGHG2	IGHG2
IGHG3	IGHG3	IGHG3
IGHG4	IGHG4	IGHG4
IGHM	IGHM	IGHM
IGHV3-15	IGHV3-15	IGHV3-15
IGHV3-30-5	IGHV3-30-5	IGHV3-30-5
IGHV3-7	IGHV3-7	IGHV3-7

IGHV3-74	IGHV3-74	IGHV3-74
IGK	IGK	IGK
IGKC	IGKC	IGKC
IGKV3-20	IGKV3-20	IGKV3-20
IGL1	IGL1	IGL1
IGLC3	IGLC3	IGLC3
IGLV2	-	IGLV2
IGLV3	IGLV3	-
IGLV3-21	IGLV3-21	IGLV3-21
IGLV3-25	IGLV3-25	IGLV3-25
IGLV7-46	IGLV7-46	IGLV7-46
IGM	IGM	IGM
ITIH1	ITIH1	ITIH1
ITIH2	ITIH2	ITIH2
ITIH3	ITIH3	ITIH3
ITIH4	ITIH4	ITIH4
JCHAIN	JCHAIN	JCHAIN
JUP	JUP	JUP
KLKB1	KLKB1	KLKB1
KNG1	KNG1	KNG1
KPRP	KPRP	KPRP
KRT17	KRT17	KRT17
KRT28	-	-
KRT31	KRT31	-
KRT34	KRT34	KRT34
KRT35	-	KRT35
KRT36	KRT36	-
-	-	KRT6A
KRT6B	KRT6B	-
KRT7	KRT7	KRT7
KRT71	KRT71	KRT71
KRT80	KRT80	KRT80
KRT82	KRT82	-
-	KRT83	KRT83
KRT85	KRT85	-
KRT86	KRT86	KRT86
KRT87P	KRT87P	-
KRTAP11-1	-	-
LBP	LBP	LBP
LCAT	LCAT	LCAT
LPA	LPA	LPA
LRG1	LRG1	LRG1
LUM	LUM	LUM
MAN1A1	MAN1A1	MAN1A1

MASP1	MASP1	MASP1
MST1L	MST1L	MST1L
ORM2	ORM2	
PGLYRP2	PGLYRP2	PGLYRP2
PON1	PON1	PON1
PON3	PON3	PON3
PRG4	PRG4	PRG4
PZP	PZP	PZP
RAB11A	RAB11A	RAB11A
-	S100A7	-
SAA4	SAA4	SAA4
SELENOP	SELENOP	SELENOP
SERPINA10	SERPINA10	SERPINA10
SERPINA3	SERPINA3	SERPINA3
SERPINA4	SERPINA4	SERPINA4
SERPINA6	SERPINA6	SERPINA6
SERPINB3	SERPINB3	SERPINB3
SERPINC1	SERPINC1	SERPINC1
SERPIND1	SERPIND1	SERPIND1
SERPINF1	SERPINF1	SERPINF1
SERPINF2	SERPINF2	SERPINF2
SERPING1	SERPING1	SERPING1
SHBG	SHBG	SHBG
-	TUBA4B	TUBA4B
TUBB4B	TUBB4B	TUBB4B
